# LINC00887 promotes GCN5-dependent H3K27cr level and CRC metastasis via recruitment of YEATS2 and enhancing ETS1 expression

**DOI:** 10.1038/s41419-024-07091-w

**Published:** 2024-09-30

**Authors:** Meijian Liao, Wendan Zheng, Yifan Wang, Mengting Li, Xiaolin Sun, Nan Liu, Jia Yao, Fuxing Dong, Qingling Wang, Yu Ma, Jie Mou

**Affiliations:** 1 Department of Pathology, Xuzhou Medical University, Xuzhou, 221004 PR China; 2 Department of Infectious Diseases and Center of Infectious Diseases and Pathogen Biology, the First Hospital of Jilin University, Changchun, 130061 PR China; 3 Key Laboratory of Organ Regeneration and Transplantation of the Ministry of Education, the First Hospital of Jilin University, Changchun, 130061 PR China; 4 Public Experimental Research Center, Xuzhou Medical University, Xuzhou, 221004 PR China; 5 School of Pharmacy, Xuzhou Medical University, Xuzhou, 221004 PR China

**Keywords:** Epigenetics, Colon cancer

## Abstract

Recent observations have revealed upregulation of H3K27cr in colorectal cancer (CRC) tissues; however, the underlying cause remains elusive. This study aimed to investigate the mechanism of H3K27cr upregulation and its roles in CRC metastasis. Clinically, our findings showed that H3K27cr served as a highly accurate diagnostic marker to distinguish CRC tissues from healthy controls. Elevated levels of LINC00887 and H3K27cr were associated with a poorer prognosis in CRC patients. Functionally, LINC00887 and H3K27cr facilitated the migration and invasion of CRC cells. Mechanistically, LINC00887 interacted with SIRT3 protein. Overexpressed of LINC00887 obstructed the enrichment of SIRT3 within GCN5 promoter, thereby elevating H3K27ac but not H3K27cr level within this region, subsequently activating GCN5 expression. This activation increased the global level of H3K27cr, promoting the enrichment of GCN5, H3K27cr, and YEATS2 within ETS1 promoter, activating ETS1 transcription and ultimately promoting the metastasis of CRC. The in vivo study demonstrated that inhibition of LINC00887 suppressed CRC metastasis, but this inhibitory effect was nullified when mice were treated with NaCr. In conclusion, our results confirmed the diagnostic biomarker potential of H3K27cr in individuals with CRC, and proposed a functional model to elucidate the involvement of LINC00887 in promoting CRC metastasis by elevating H3K27cr level.

## Introduction

H3K27cr, the crotonylation of histone H3 at lysine 27, is a crucial factor in the direct regulation of gene transcription in human health and disease. In physiological processes, such as gametogenesis, H3K27cr has been identified as a novel marker at the promoters and distal enhancers of active genes [1]. In cancer progression, our previous research has shown that H3K27cr is not only linked to DNA damage, but also facilitates cell invasion and migration [2, 3]. Moreover, H3K27cr is specifically recognized by GAS41, leading to the regulation of cell proliferation and cell-cycle arrest by modulating p21 expression [4]. The mutation of YEATS domain, which eliminates GAS41 binding to H3K27cr, leads to nuclear shape abnormalities and cell proliferation inhibition [3, 5]. In other disease, such as type 2 diabetes mellitus, H3K27cr is involved in the regulation of glucose uptake by inhibiting GLUT4 transcription [6]. Similarly, in Alzheimer’s disease, H3K27cr regulates the uptake and degradation of Aβ through the modulation of endocytosis-related gene expression [7].

Our previous study shows that H3K27cr level is elevated in CRC tissues and correlates with the tumor-node-metastasis (TNM) stage of CRC patients [2]. However, the underlying reason remains unclear. Recently, reports show that histone crotonylation is regulated by crotonyltransferases and decrotonylases. GCN5, traditionally knowned as an acetyltransferase, may function as a histone crotonyltransferase, as deletion of GCN5 reduces histone crotonylation [8]. GCN5 is highly expressed in various cancers, including CRC [9], lung cancer [10], hepatocellular carcinoma [11], and human glioma [12]. Furthermore, other crotonyltransferases, such as P300, are elevated in CRC tissues as well [13]. This could be attributed to crotonyltransferase overexpression in cancerous tissues, elevating H3K27cr level. However, additional research is required to investigate this further.

Histone crotonylation is widely acknowledged as an active marker [14]. Recent evidences have demonstrated that histone crotonylation activates gene transcription via its readers [1, 15]. The YEATS domain of readers exhibit an affinity for the planar crotonylamide group and effectively encloses it between two aromatic residues, thereby establishing an “aromatic-π-aromatic” stacking configuration [16–18]. Ultimately, this molecular interaction causes positive modulation of gene transcription. However, some studies demonstrate that GAS41 specifically recognizes H3K27cr, leading to the recruitment of SIN3A-HDAC1 co-repressors and repression of gene transcription[4]. The specific reader that recognizes crotonylation determines the promotion or inhibition of gene transcription. Nevertheless, the mechanisms by which distinct readers are selected for histone crotonylation have yet to be fully understood.

Previous research has posited that histone crotonylation marks are predominantly found at promoter or enhancer sites [14]. However, the mechanisms underlying the specific aggregation of histone crotonylation at particular chromosomal positions remain elusive. Recent investigations have proved that lncRNAs interact with crotonylation-modifying enzymes and regulate their recruitment to the chromatin, suggesting that lncRNAs may regulate the enrichment of crotonylation at distinct chromatin positions. For example, lncRNAs, such as NEAT1 or EPB41L4A-AS1, interact with crotonyltransferases, such as P300/CBP complex or GCN5, to regulate H3K27cr level at a specific location [6, 7].

LINC00887, a lncRNA, has been controversial regarding its role in tumorigenesis and tumor development. LINC00887 facilitates cell proliferation in glioma, clear cell renal cell carcinoma, nasopharyngeal carcinoma, and lung carcinoma [19–22]. However, it is contradictory that LINC00887 suppresses cervical cancer cell proliferation and invasion [23]. Presently, there is a lack of information regarding the role of LINC00887 in CRC. Our study revealed that LINC00887 was elevated in CRC tissues, which was associated with unfavorable survival outcomes in CRC patients. LINC00887 overexpression facilitated the recruitment of GCN5 to ETS1 promoter, leading to H3K27cr enrichment and recognition by YEATS2. Ultimately, this event cascade resulted in enhanced ETS1 transcription and CRC metastasis. These findings supported the roles of LINC00887 and H3K27cr in promoting CRC metastasis.

## Materials and methods

### Cell culture

The human colorectal cancer cell lines HCT116 (TCHu 99) and LoVo (TCHu 82) were procured from the Cell Bank of the Chinese Academy of Sciences (Shanghai, China). HCT116 and LoVo cells were cultured in Dulbecco’s Modified Eagle Medium (DMEM) (Bio-Channel, Cat. No. BC-M-005, Nanjing, China) supplemented with 10% fetal bovine serum (FBS) (ExCell Bio, Cat. No. FSP500, Suzhou, China) and 1% antibiotic solution (Bio-Channel, Cat. No. BC-CE-007, Nanjing, China). Cells were maintained at 37 °C in a humidified incubator with 5% CO_2_.

### Datasets

The transcription profiles of epithelial cells obtained by laser microdissection in the GSE15960 dataset and the single-cell transcriptome profiles of GSE110009 and GSE163974 datasets were downloaded from the gene expression omnibus (GEO) database. The transcription profiles and clinical information for colorectal adenocarcinoma (COAD) in the cancer genome atlas (TCGA) dataset were downloaded from the UCSC Xena database. The gene set representing the epithelial-mesenchymal transition (EMT) pathway was downloaded from the molecular signatures database (MSigDB) database. The ChIP-seq data of GSE128590 was provided by Nan Liu.

### Transfection and lentiviral transduction

SiRNAs and plasmids were acquired from Integrated Biotech Solutions (IBSBIO, Shanghai, China) and Genechem Co. Ltd. (Shanghai, China). SiRNAs and plasmids were transfected using Lipofectamine 2000^TM^ (Invitrogen, Cat. No. 11668-027, USA) and lipid transfection reagent (YEASEN, Cat. No. 40802ES08; Shanghai, China). Lentivirus containing sh-LINC00887 was procured from OBIO Technology (Shanghai, China). Stable knockdown cells were established by infecting HCT116 cells with sh-LINC00887 lentivirus and selecting 4 μg/mL puromycin (Beyotime, Cat. No. ST551-10 mg, Shanghai, China) for 14 days. The siRNA sequences are listed in Supplementary Table S1.

### ScRNA-seq analysis

Single-cell transcriptome profiles were analyzed using the Seurat package. FindIntegrationAnchors and IntegrateData functions were employed to eliminate batch effects across each sample. Subsequently, FindClusters and RunUMAP functions were executed to determine the clustering of each cell, with a resolution of 1.5. Marker genes for each subtype were identified using the FindAllMarkers function, and the cell types were annotated using CellMarker 2.0. The subset function was used to extract tissue sources from each cell. The fgseaMultilevel function from the fgsea package was used to compare gene sets for each type of cell, and the outcomes were visualized using the ggplot2 package. The R code of scRNA-seq analysis was referred to by Liao et al. [2], and the key parameters were available in the supplementary material.

### Gene set enrichment analysis (GSEA)

The GSEA software (version 4.3.2) has a limitation in analyzing gene sets with fewer than 500 genes. Therefore, a total of 158 genes with at least three peaks of H3K27cr occupancy in their promoters were identified as the H3K27cr ChIP-seq gene set by Liao et al. [2]. Additionally, 455 genes with at least four peaks of H3K27cr occupancy in their promoters were identified as the H3K27cr ChIP-seq gene set by Liu et al. [4]. The COAD tissues in TCGA dataset and epithelial cells in GSE15960 dataset were divided into two groups based on the median level of each lncRNA. One thousand permutations were used for each gene set. The results with false discovery rate (FDR) < 0.25 and *P* < 0.05 were considered significant enrichment.

### Weighted gene co-expression network analysis (WGCNA)

Gene co-expression modules of epithelial cells in the GSE15960 dataset were determined using the WGCNA package. To assess the relationship between CRC and each module, normal tissues were assigned as 1, adenoma tissues as 2, and CRC tissues as 3, followed by correlation analysis using the COR function. Enrichment analysis of gene sets associated with EMT or H3K27cr ChIP (LINC00887 vs. control) in each module was performed using the Fisher test, and significance was evaluated using the hypergeometric test. The R code for the WGCNA analysis was referenced from Wu et al. [24].

### Western blotting

Total protein was extracted from the cells with RIPA lysis buffer (Beyotime, Cat. No. P0013B, Shanghai, China) supplemented with a fresh protease and phosphatase inhibitor cocktail. The protein concentration was assessed using a BCA assay kit (KeyGEN BioTECH, Cat. No. KGB2101-1000, Nanjing, China). Subsequently, 10% SDS-PAGE was conducted to segregate the proteins based on their molecular weights, followed by transferring the separated proteins onto a cellulose nitrate membrane. Then, the membranes were blocked with 5% skim milk for 2 h and incubated overnight at 4 °C with primary antibodies. The next day, the membranes were incubated with secondary antibodies for 1 h. The following antibodies were used: anti-E-Cadherin (1:2,000, ABclonal, Cat. No. A20798), anti-N-Cadherin (1:1,000, ABclonal, Cat. No. A3045), anti-vimentin (1:1,000, ABclonal, Cat. No. A19607), anti-ETS1 (1:1,000, Beyotime, Cat. No. AF6812), anti-SIRT3 (1:200; Santa Cruz, Cat. No. SC-365175), anti-GCN5 (1:1,000, Affinity, Cat. No. DF3383), anti-H3K27cr (1:1,000, PTM BIO, Cat. No. PTM-545PM), anti-GAPDH (1:10,000, Proteintech, Cat. No. 60004-1-Ig), anti-Histone H3 (1:10,000, Abbkine, Cat. No. ABL1070), anti-rabbit IgG (1:10,000, SAB, Cat. No. L3012), and anti-mouse IgG (1:10,000, SAB, Cat. No. L3032).

### Quantitative real-time polymerase chain reaction (qRT-PCR)

Total RNA was extracted from cells or CRC tissues using TRIzol (Vazyme, Cat. No. R401-01, Nanjing, China). Subsequently, RNA was reverse-transcribed using the SweScrip RT I First Strand cDNA Synthesis Kit (ServiceBio, Cat. No. G3330, Wuhan, China). The qRT-PCR analysis was performed using an ABI StepOne Real-Time PCR System (Carlsbad, USA) and 2×SYBR Green qPCR Master Mix (High ROX) (Servicebio, Cat. No. G3332, Wuhan, China). GAPDH was employed to ensure gene normalization, and the relative fold changes were determined using the 2^−ΔΔCt^ method. The primer sequences for qRT-PCR are illustrated in Supplementary Table S2. The qRT-PCR assay for CRC tissues was approved by the Ethics Committee of Shanghai Outdo Biotech Company (SHYJS-CP-1704001).

### Transwell assay

In this study, improved Transwell chambers with 8.0 μm pores (BD Bioscience, Cat. No. 3422, San Jose, USA) and Transwell inserts with or without Matrigel coating (BD Biosciences, Cat. No. 356234, San Jose, USA) were used to evaluate the migratory and invasive abilities of cells. In a bicameral culture system, 200 µL of serum-free medium containing transfected suspension cells was added to the upper chamber, and 600 µL of medium supplemented with 10% FBS was added to the lower chamber. After 24 h of migration culture and 48 h of invasion culture, cells successfully traversed the pores and were immobilized using a 4% paraformaldehyde solution (Vicmed, Cat. No. VIH100, Xuzhou, China). Following this, the cells were stained with 1% crystal violet (Vicmed, Cat. No. VS1003, Xuzhou, China). Images were acquired using an Olympus microscope at a magnification of ×80, and the cells that successfully penetrated the pores were quantified using the ImageJ software. All experiments were performed in triplicate to ensure reliable statistical analyses.

### Chromatin immunoprecipitation (ChIP-seq and ChIP-qPCR) analysis

The cells were cross-linked with 1% formaldehyde and re-suspended in ChIP lysis buffer containing 150 mM NaCl, 5 mM EDTA, 0.1% deoxycholic acid, 1% Triton X-100, 50 mM Tris-HCl (pH 8.0), and 20 μL/mL protease inhibitor cocktail. Afterward, the cell lysates were sonicated and centrifuged. The supernatant was incubated with 2 μg of antibody and 25 μL of protein A/G beads (MedChemExpress, Cat. No. HY-K0202, USA) for 4 h at 4 °C. ChIP assays were conducted using antibodies targeting H3K27ac, SIRT3, YEATS1, GCN5, and H3K27cr, with rabbit IgG (Beyotime, Cat. No. A7016, Shanghai, China) and mouse IgG (Beyotime, Cat. No. BD0050, Shanghai, China) served as control antibodies. Following three washes, DNA samples were purified and analyzed using qRT-PCR or sequencing by Orizymes (Shanghai, China).

### RNA immunoprecipitation (RIP)

The cells were collected and digested with RIP lysis buffer containing 5 mM MgCl_2_, 10 mM HEPES (pH 7.0), 10 mM KCl, 1 mM DTT, 100 U/mL RRI, 0.5% NP-40, 20 µL/mL protease inhibitor, and 2 mM vanadyl ribonucleotide complex solution. Subsequently, 3 μg of antibody was added to the cell lysates and incubated overnight at 4 °C, with IgG antibody as a control. Then, protein A/G beads were introduced to the reaction mixture and incubated for 4 h at 4 °C. Following three washes, the RNA bound to the beads was purified using TRIZOL, and its presence was determined using qRT-PCR. The following antibodies were used: anti-SIRT1 (Proteintech, Cat. No. 13161-1-AP); anti-SIRT2 (Proteintech, Cat. No. 19655-1-AP); anti-SIRT3 (Santa Cruz, Cat. No. SC-365175); anti-SIRT5 (Santa Cruz, Cat. No. SC-271635); anti-SIRT6 (Proteintech, Cat. No. 13572-1-AP); anti-GCN5 (Affinity, Cat. No. DF3383); anti-HDAC2 (Santa Cruz, Cat. No. SC-9959); anti-P300 (Santa Cruz, Cat. No. SC-48343).

### Fluorescent in situ hybridization (FISH) and immunofluorescence

The LINC00887 RNA probe, labeled with Cyanine 5, was obtained from Integrated Biotech Solutions (Shanghai, China). Briefly, cells were seeded onto 12-well coverslips, and the coverslips were washed with PBS five times on the second day. Subsequently, the cells were permeabilized with cytoskeletal buffer [100 mM NaCl, 300 mM sucrose, 3 mM MgCl2, 10 mM PIPES, pH 6.8] on ice for 5 min and fixed with 4% paraformaldehyde for 10 min. Following fixation, cells were dehydrated with 80%, 95%, and 100% ethanol for 3 min at each concentration. Then, the LINC00887 probe was applied to the cells and incubated at 37 °C overnight in a dark and humid chamber. The coverslips were washed three times with freshly prepared 50% formamide and 2× SSC buffer at 42 °C for 5 min each. Following this, cells were blocked using a 1% BSA solution for 15 min, exposed to the SIRT3 antibody (1:50) for 45 min, and incubated with Alexa fluor 488-conjugated affiniPure goat anti-mouse IgG (H + L) (Beyotime, Cat. No. A0428, Shanghai, China) for 1 h. Finally, the specimens were counterstained with DAPI and mounted for examination using a STELLARIS 5 confocal fluorescence microscope (Leica).

### Immunohistochemistry (IHC)

The CRC tissue chip (ID: HColA160Su02), consisting of 100 cases of CRC tissues and 60 cases of para-cancer tissues, was procured from Shanghai Otu Biotech Co. Ltd. (Shanghai, China). The tissue sections were deparaffinized at 65 °C for 1.5 h, followed by re-deparaffinization in xylene. Antigens were retrieved by heating sections in boiling 0.01 M citrate buffer for 10 min. Tissue microarrays were blocked with 1% normal goat serum for 2 h to minimize non-specific staining. Then, the tissues were incubated with the primary antibody at 4 °C overnight in a humid chamber. After washing with PBS, the tissues were individually incubated with the secondary antibody and stained using a DAB detection kit (Zhongshan Biotech, Cat. No. ZLI-9018, Beijing, China). Lastly, the tissues were counterstained with hematoxylin and sealed with neutral resin before meticulous examination under a microscope by two observers.

The results revealed that 3 tissues labeled as “cancer” did not contain cancer cells, and 2 tissues labeled as “para-cancer” lacked epithelial tissue. This may be attributed to tissue detachment during the IHC assay. Consequently, we analyzed the levels of H3K27cr levels in 97 cases of cancer tissues and 58 cases of para-cancer tissues. The H3K27cr level was assessed using the Allred score [25]. The staining intensity was graded on a scale of 0 to 3, with 0 indicating no staining, 1 indicating weak staining, 2 indicating moderate staining, and 3 indicating strong staining. The extent of positive cell area was converted into a grading system ranging from 0 to 5, with specific ranges assigned to each grade: 0 (<1%), 1 (1%–10%), 2 (11%–50%), 3 (51%–80%), and 4 (>81%). Additionally, two grades yielded a score ranging from 0 to 7, with H3K27cr levels greater than 5 considered high. Kaplan–Meier survival curves were employed to evaluate the association between H3K27cr levels and the survival of patients with CRC. IHC assays for CRC tissues were approved by the Ethics Committee of the Shanghai Outdo Biotech Company (SHYJS-CP-1704001).

### Animal experiment

The animal experiments conducted in this study were approved by the Institutional Animal Care and Use Committee (IACUC) of Xuzhou Medical University (NO: 202306T018), China. Five-week-old BALB/c nude mice were obtained from GemPharmatech (Nanjing, China) and were randomly assigned to four groups (A, B, C, and D) with 9 mice in each group. Mice in groups C and D were administered intraperitoneal injections of NaCr at a dosage of 5.2 mg • kg^–1^ daily. After three days, mice in groups B and D were injected with HCT116 cells with stable knockdown of LINC00887 via the tail vein, with 2 × 10^6^ cells per injection. Conversely, mice in groups A and C were injected with control cells via the tail vein at 2 × 10^6^ cells per injection. Mice in groups C and D received additional injections of NaCr at a dosage of 5.2 mg • kg^–1^ every two days. Seven weeks after injection, all mice were euthanized, and their lung tissues were collected to quantify the metastatic nodules in each mouse. Finally, the lung tissues underwent HE and IHC analyses.

### Statistical analysis

The statistical analysis was conducted using GraphPad Prism software (version 8.0), and the graphs were generated using the same software. Differences between the two groups were assessed using the Mann–Whitney U test, paired or unpaired Student’s *t* test, or chi-square test. Given that H3K27cr level was discontinuous variables, the association between LINC00887 expression and H3K27cr level was examined using Spearman rather than Pearson correlation analysis. The distribution of LINC00887 between the nucleus and cytoplasm was analyzed using the chi-square test. Prognosis analysis was conducted using the Kaplan–Meier survival curve analysis. All experiments were independently performed three times, and the data were presented as means ± standard deviation. Statistical significance was denoted as *P < 0.05, **P < 0.01, ***P < 0.001, ****P < 0.0001.

## Results

### High level of H3K27cr is associated with poor prognosis

**Fig. 1:**
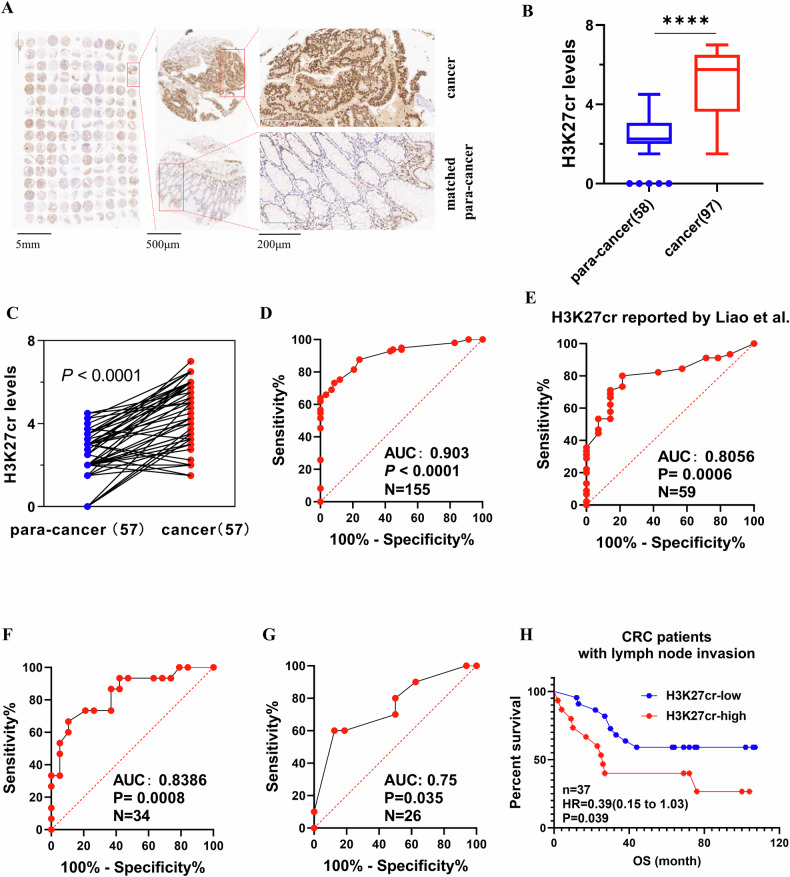
A High level of H3K27cr is associated with poor prognosis. **A** Representative immunohistochemical images of H3K27cr level between CRC and matched para-cancer tissues. Mann–Whitney U analysis (**B**) and paired *t*-test (**C**) analyzing H3K27cr levels between CRC and para-cancer tissues. ****P < 0.0001. ROC curve analysis of H3K27cr level to distinguish CRC patients from healthy controls (**D**, **E**), CRC patients with distant metastasis from those without (**F**), metastatic sites of CRC tissues from primary CRC tissues (**G**). **H** Kaplan–Meier survival curve analysis of the relationship between H3K27cr level and overall survival (OS) of CRC patients with lymph node invasion (*n* = 37).

We detected H3K27cr level in CRC tissues from 97 patients and para-cancer tissues from 58 patients. The results revealed that cancerous tissues exhibited higher level of H3K27cr than normal tissues (Fig. 1A–C, Table 1). We investigated the potential diagnostic utility of H3K27cr for differentiating CRC tissues from healthy controls. Notably, receiver operating characteristic (ROC) curve analysis revealed an area under the curve (AUC) value of 0.9 (Fig. 1D). Furthermore, this analysis was replicated in a cohort of our previous study [2], yielding an AUC value of 0.81 (Fig. 1E). We also evaluated the diagnostic value of H3K27cr in distinguishing CRC patients with distant metastasis from those without, resulting in an AUC value of 0.84 (Fig. 1F). Furthermore, H3K27cr demonstrated the capability to differentiate metastatic sites from primary CRC tissues, with an AUC value of 0.75 (Fig. 1G). Notably, among CRC patients with lymph node invasion, those with elevated level of H3K27cr exhibited a poorer prognosis (Fig. 1H).

**Table 1:** H3K27cr levels between cancer and para-cancer tissues.

	H3K27cr level		*n*	Chi-square value	*p*-value
	Low	High			
Cancer	44	53	97	48.16	< 0.001
Para-cancer	58	0	58		

### LINC00887 elevates H3K27cr level

**Fig. 2:**
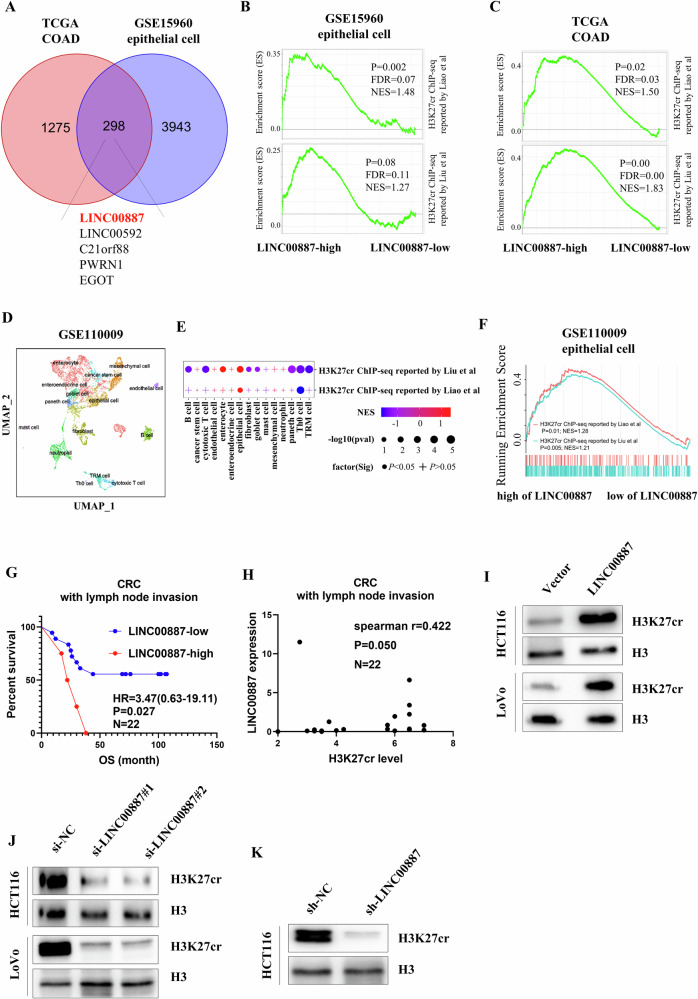
LINC00887 elevates H3K27cr level. **A** Overlay of genes differentially expressed in COAD tissues from TCGA database and in epithelial cells from the GSE15960 dataset. Analysis of genes with promoters occupied by H3K27cr in epithelial cells (**B**) or in COAD tissues (**C**) with LINC00887 high or low expression. **D** UMAP analysis of single-cell transcriptome profiles of CRC tissues from the GSE110009 dataset. Colors represent different cell subtypes. Fgsea analyzes the enrichment of genes with promoters occupied by H3K27cr across different cell types (**E**), or epithelial cells with high or low LINC00887 expression (**F**). **G** Kaplan–Meier survival curve analysis of the prognosis of CRC patients with high or low expression of LINC00887. **H** Spearman’s correlation analysis of the association between H3K27cr level and LINC00887 expression in CRC tissues (*n* = 22). **I**–**K** Analysis of H3K27cr level in CRC cells with LINC00887 instantaneous overexpression (**I**), instantaneous knockdown (**J**) or stable knockdown (**K**). The H3 acts as a loading control.

Next, we investigated whether lncRNAs regulated H3K27cr modification. We identified genes with differential expression in colon adenocarcinoma (COAD) from the TCGA dataset or in epithelial cells of CRC patients obtained using laser microdissection (GSE15960). The findings revealed that 1573 and 4241 genes were differential expression in COAD tissues and epithelial cells derived from CRC tissues, respectively (|logFC | >1, *P* < 0.05, Supplementary Fig. S1A, B). Combining these two results revealed 298 differentially expressed genes, including five lncRNAs (Fig. 2A). The association between these five lncRNAs and genes with promoters occupied by H3K27cr was examined using GSEA analysis. These findings demonstrated that genes with promoters occupied by H3K27cr were significantly enriched in epithelial cells or COAD tissues with high LINC00887 expression (Fig. 2B, C and Supplementary Fig. S1C–F). Subsequently, a similar analysis was conducted on epithelial cells derived from the single-cell transcription profiles. A total of 8,083 cells from the GSE110009 dataset were examined, identifying 15 distinct cell subtypes (Fig. 2D and Supplementary Fig. S1G). Genes with promoters occupied by H3K27cr were enriched in epithelial and Th0 cells (Fig. 2E). A GSEA result revealed a significant enrichment of genes with promoters occupied by H3K27cr in epithelial cells with high LINC00887 expression but not in Th0 cells (Fig. 2F and Supplementary Fig. S1H). These findings suggested a positive association between LINC00887 level and H3K27cr-regulating genes.

LINC00887 expression was upregulated in COAD tissues (Supplementary Fig. S2A). It was observed an elevated LINC00887 expression in advanced cancer or cancer tissues with lymph node invasion (Supplementary Fig. S2B, C). High LINC00887 expression was significantly associated with poor prognosis among COAD patients (Supplementary Fig. S2D). Next, we conducted qRT-PCR to detect LINC00887 expression in CRC patients with lymph node invasion. The results indicated that high LINC00887 expression significantly correlated with poor prognosis (Fig. 2G). Spearman’s correlation analysis demonstrated a positive association between LINC00887 and H3K27cr levels (Fig. 2H). LINC00887 overexpression increased H3K27cr level (Fig. 2I and Supplementary Fig. S2E). Conversely, transient and stable LINC00887 knockdown had the opposite effect (Fig. 2J, K and Supplementary Fig. S2F-G). These findings provided evidence that LINC00887 promoted H3K27cr level.

### LINC00887 elevates H3K27cr level by inducing GCN5 expression

**Fig. 3:**
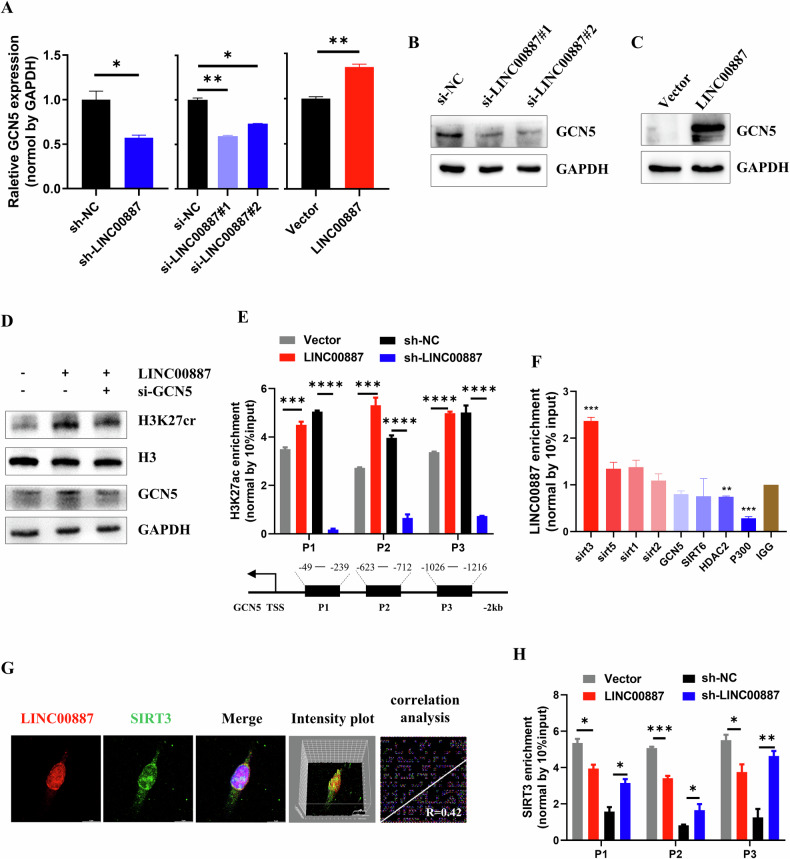
LINC00887 elevates H3K27cr level by activating GCN5 expression. **A** qRT-PCR analysis of GCN5 expression in HCT116 cells with stable or instantaneous knockdown of LINC00887, or LINC00887 overexpression. Immunoblotting analysis of GCN5 level in HCT116 cells transfected with siLINC00887 (**B**) or LINC00887 plasmid (**C**) for 48 h. GAPDH served as the loading control. **D** Immunoblotting analysis of H3K27cr level in HCT116 cells co-transfected with siGCN5 and LINC00887 plasmid for 48 h. The H3 and GAPDH served as loading controls. **E** The ChIP-PCR assay analysis of the enrichment of H3K27ac across GCN5 promoter in HCT116 cells with instantaneous overexpression or stable knockdown of LINC00887. **F** The RIP assay analysis of the interaction between SIRT3 and LINC00887 in HCT116 cells. **G** RNA-FISH showed the co-localization between SIRT3 and LINC00887 in HCT116 cells. Scale bar, 9 μm. The intensity plot and correlation analysis were finished by image J software. **H** The ChIP-PCR assay analysis of the enrichment of SIRT3 across GCN5 promoter in HCT116 cells with instantaneous overexpression or stable knockdown of LINC00887. Data are represented as means ± SD, *P < 0.05, **P < 0.01, ***P < 0.001, ****P < 0.0001, unpaired, two-tailed, Student’s *t* test.

We next investigated the mechanism of LINC00887 increasing H3K27cr. GCN5, a histone crotonyltransferase [26], is significantly elevated in CRC tissues (Supplementary Fig. S3A, B). It was observed that stable and transient LINC00887 knockdown decreased GCN5 expression, whereas LINC00887 overexpression enhanced GCN5 level (Fig. 3A–C). Furthermore, a rescue experiment involving co-transfection of LINC00887 plasmid and siGCN5 restored H3K27cr level (Fig. 3D). Subsequently, we studied the alteration of histone H3K27 modification of GCN5 promoter. The results revealed that LINC00887 overexpression elevated H3K27ac level, but not H3K27cr level, across this region (Fig. 3E and Supplementary Fig. S3C). To investigate the molecular mechanism how LINC00887 influenced H3K27ac enrichment on GCN5 promoter, we initially examined the localization of LINC00887 in CRC cells, revealing predominant nuclear localization (Figs. S3D-E). Therefore, we hypothesized that whether LINC00887 regulated H3K27ac enrichment on the GCN5 promoter by interacting with acetylation-related enzymes. Subsequently, a RIP assay was conducted to identify proteins that interacted with LINC00887. The result revealed a significant interaction between SIRT3 and LINC00887 (Fig. 3F). This interaction was further confirmed using RNA-FISH assay, showing their co-localization in the nucleus (Fig. 3G). It was also observed that LINC00887 overexpression reduced SIRT3 enrichment in the GCN5 promoter, whereas LINC00887 knockdown increased (Fig. 3H). These findings suggested that LINC00887 interacted with SIRT3. Its overexpression impeded the binding of SIRT3 to GCN5 promoter, elevating H3K27ac level in this region and activating GCN5 transcription, ultimately increasing H3K27cr level.

### H3K27cr regulates cell adhesion of epithelial cells derived from CRC tissues

**Fig. 4:**
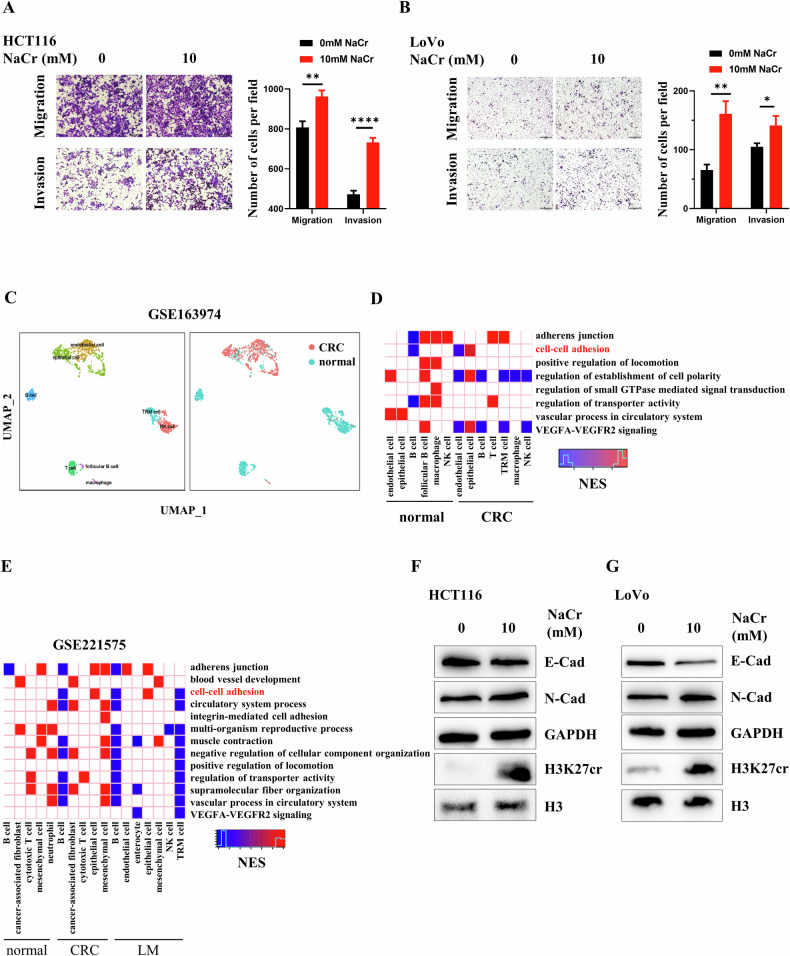
H3K27cr regulates cell adhesion of epithelial cells derived from CRC tissues. Representative images of invasion and migration of HCT116 (**A**) and LoVo (**B**) cells treated with 10 mM NaCr for 48 h. Scale bar, 200 μm. The cell numbers were calculated in three random fields (right panel). Data are presented as mean ± SD, *P < 0.05, **P < 0.01, ****P < 0.0001, unpaired, two-tailed, Student’s *t* test. **C**. UMAP plotting the dis*t*ribution of different cells across CRC tissues (GSE163974). The colors in the left and right panels represent the different cell and tissue types, respectively. Enrichment of pathways across different subtypes of cells derived from normal and CRC tissues in the GSE163974 (**D**) and GSE221575 (**E**) datasets. Colors represent the values of the normalized enrichment score (NES). Immunoblotting analysis of E-cadherin, N-cadherin, and H3K27cr levels in HCT116 (**F**) and LoVo (**G**) cells treated with 10 mM NaCr for 48 h. GAPDH and H3 served as loading controls.

We studied the mechanism of H3K27cr in regulating invasion. We treated CRC cells with NaCr and observed an elevation of invasion and migration (Fig. 4A, B). Following, we analyzed the functional pathway of genes with promoters occupied by H3K27cr reported by Liao et al. [2]. 20 pathways were enriched, including the cell-cell adhesion pathway (Supplementary Fig. S4A). Subsequently, scRNA-seq analysis was conducted to assess the presence of these 20 pathways in specific cell types. A total of 1,688 cells were analyzed in the GSE163974 dataset, identifying eight distinct cell subtypes (Fig. 4C and Supplementary Fig. S4B). By comparing 20 pathways across various cell subtypes, it was observed that cell-cell adhesion was specific enriched in epithelial cells derived from CRC tissues (Fig. 4D). This result was further confirmed in GSE221575, a previously analyzed dataset (Fig. 4E) [2]. CRC cells treatment with NaCr reduced E-cadherin, while inducing N-cadherin (Fig. 4F, G). This finding implied that H3K27cr might play a regulatory role in CRC metastasis via its impact on cell adhesion.

### LINC00887 facilitates the invasion and migration of CRC cells

**Fig. 5:**
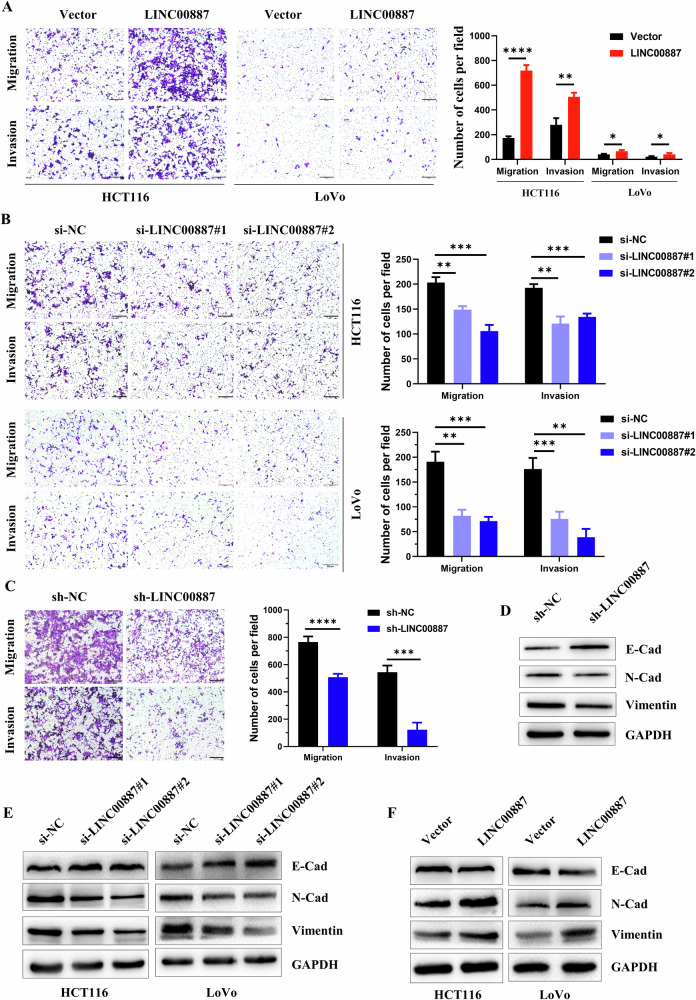
LINC00887 promotes the invasion and migration of CRC cells. Representative images of invasion and migration levels in CRC cells with LINC00887 instantaneous overexpression (**A**) and knockdown (**B**) for 48 h, and LINC00887 stable knockdown (**C**). Scale bar, 200 μm. The cell numbers were calculated in three random fields (right panel). Data are presented as mean ± SD, *P < 0.05, **P < 0.01, ***P < 0.001, ****P < 0.0001, unpaired, two-tailed, Student’s *t* test. Immunoblotting analysis of E-cadherin, N-cadherin, and vimentin levels in CRC cells with LINC00887 stable (**D**), instantaneous knockdown (**E**), and instantaneous overexpression (**F**) for 48 h. GAPDH served as the loading control.

Considering the promotion of H3K27cr level by LINC00887, we studied the impact of LINC00887 on the acceleration of invasion and migration. The findings revealed that LINC00887 overexpression increased invasion and migration levels in CRC cells, whereas immediate and sustained LINC00887 knockdown diminished these levels (Fig. 5A–C). Additionally, immediate and sustained LINC00887 knockdown upregulated E-cadherin expression and downregulated N-cadherin and vimentin, whereas LINC00887 overexpression had the opposite effect on these markers (Fig. 5D–F). These findings suggested that LINC00887 facilitated the invasion and migration of CRC cells.

### LINC00887 facilitates invasion and migration via the H3K27cr-mediated cell adhesion molecules (CAMs)

**Fig. 6:**
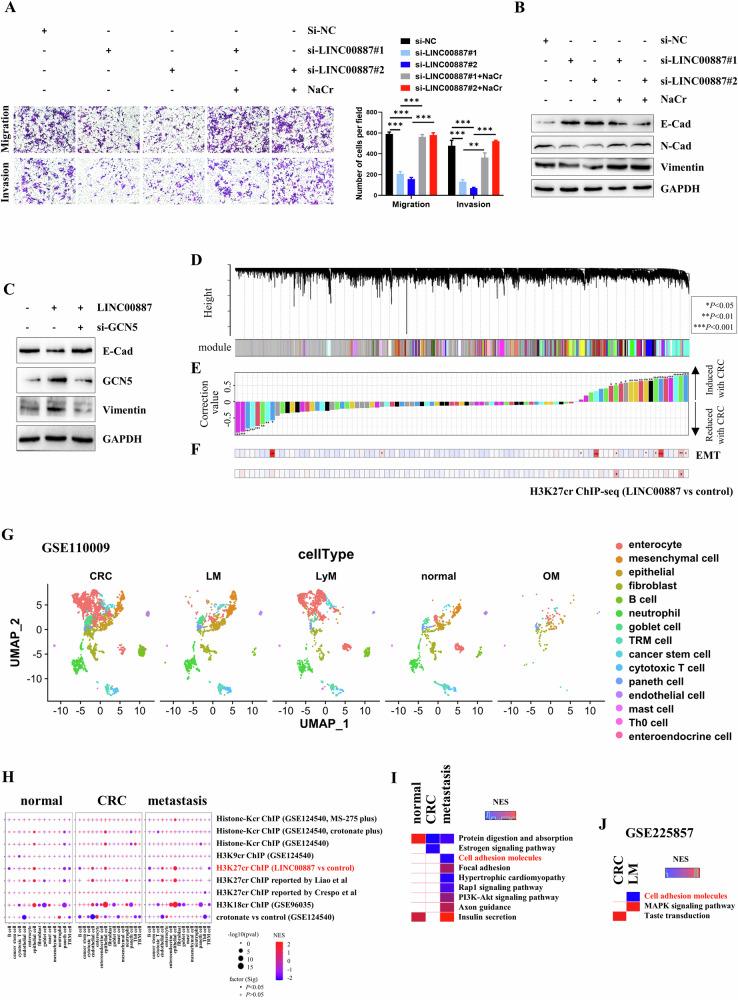
LINC00887 promotes the invasion and migration of CRC cells by regulating H3K27cr-mediated CAMs. **A** Representative image analysis of invasion and migration levels in HCT116 cells transfected with siLINC00887 and supplemented with NaCr for 48 h. Scale bar, 200 μm. The cell numbers were calculated in three random fields (right panel). Data are presented as mean ± SD, **P < 0.01, ***P < 0.001, unpaired, two-tailed, Student’s *t* test. **B**, **C** Immunoblo*t*ting analysis of E-cadherin, N-cadherin, and vimentin levels in HCT116 cells transfected with siLINC00887 and supplemented with NaCr for 48 h (**B**) or HCT116 cells co-transfected with siGCN5 and LINC00887 plasmid for 48 h (**C**). The GAPDH served as loading control. **D** WGCNA dendrogram presenting co-expression modules in epithelial cells obtained through laser microdissection (GSE15960). The colors represent different modules. **E** Spearman’s correlation evaluating the CRC-associated modules. **F** Enrichment analysis of EMT pathway genes (upper panel) and 602 genes (lower panel) across various modules. The *P*-value was calculated using a hypergeometric test. **G** The UMAP plot shows the distribution of cells in the GSE110009 dataset. Colors represent different cell types. OM omentum metastasis; LyM lymph node metastasis; LM liver metastasis. **H** The dot plot shows the enrichment of 602 genes and other histone crotonylation-regulated gene sets across different cell types. Colors represent the NES values. Heatmap showing the enrichment of pathways annotated by 602 genes in epithelial cells extracted from primary CRC and metastatic tissues in the GSE110009 (**I**) or GSE225857 datasets (**J**). Colors represent the values of the NES. The metastatic tissues in Fig. 6H, I include OM, LyM, and LM tissues.

Then, we investigated whether LINC00887 regulated the invasion and migration via H3K27cr. We conducted a rescue assay in HCT116 cells with siLINC00887 transfection and 10 mM NaCr treatment. The results revealed that the restoration of H3K27cr level with NaCr treatment led to the restoration of invasion and migration levels, as well as the levels of E-cadherin, N-cadherin, and vimentin (Fig. 6A, B and Supplementary Fig. S5A). Additionally, a rescue assay demonstrated that GCN5 knockdown restored the E-cadherin and vimentin levels regulated by LINC00887 (Fig. 6C), indicating that LINC00887 regulated the invasion and migration of CRC cells via the GCN5/H3K27cr pathway.

We conducted a ChIP-seq assay using an H3K27cr antibody in HCT116 cells transfected with or without the LINC00887 plasmid. The result found 17,740 differential occupancy peaks (|fold-change|> 1.2, *P* < 0.05) in response to LINC00887 overexpression, with 27.96% located in the promoter region (Supplementary Fig. S5B, Supplementary Table S3). Enrichment analysis of the genes annotated by these 17,740 peaks revealed that the adherens junction pathway had the highest enrichment score (Supplementary Fig. S5C). 602 genes exhibited at least two differential peaks within the promoter region. Next, 91 co-expression modules were identified in epithelial cells derived from normal and CRC tissues using WGCNA (Fig. 6D). Within these modules, genes in eight modules exhibited reduced expression in CRC-derived epithelial cells, while 16 modules increased (Fig. 6E). Genes in 10 modules were significantly associated with EMT pathway genes. Two of them showed a strong association with the 602 genes (Fig. 6F).

We conducted a mapping analysis of above 602 genes in single cells of the GSE110009 datase. The analysis revealed that they were significantly enriched in B cells and epithelial cells across normal, CRC, and metastatic tissues (Fig. 6G, H). Subsequently, the biological pathways of these 602 genes were annotated. The results indicated that 83 pathways contained a minimum of five genes (Supplementary Fig. S5D). Comparing these 83 pathways in epithelial cells revealed that CAMs exhibited a specific enrichment in epithelial cells derived from metastatic tissues (Fig. 6I). Notably, this result was confirmed in GSE225857 dataset, which was previously analyzed (Fig. 6J) [2]. These findings suggested that LINC00887 modulated the CAMs pathway in epithelial cells derived from metastatic tissues via H3K27cr.

### LINC00887 promotes ETS1 expression via elevating YEATS2 recruitment

**Fig. 7:**
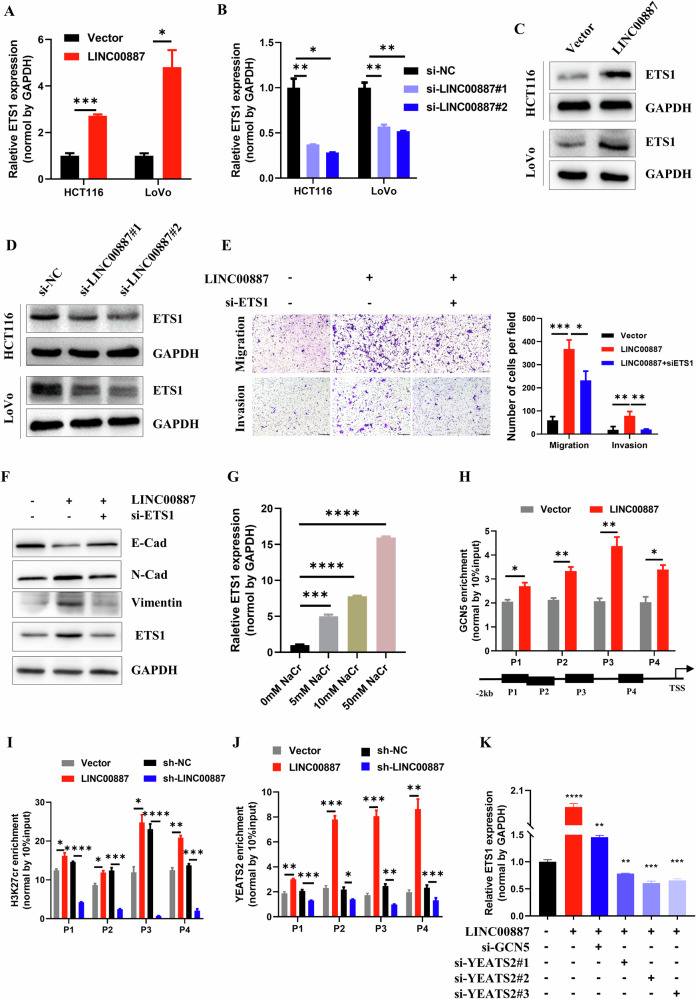
LINC00887 regulates ETS1 expression by elevating YEATS2 recruitment. qRT-PCR assay (**A**, **B**) and immunoblotting (**C**, **D**) analyses of ETS1 expression in HCT116 cells transfected with siLINC00887 or LINC00887 plasmid for 48 h. **E** Representative image analysis of invasion and migration levels in HCT116 cells co-transfection with LINC00887 plasmid and siETS1. Scale bar, 200 μm. The cell numbers were calculated in three random fields (right panel). **F** Immunoblotting analysis of E-cadherin, N-cadherin, and vimentin levels in HCT116 cells co-transfected with siETS1 and LINC00887 plasmid for 48 h. GAPDH served as the loading control. **G** qRT-PCR analysis of ETS1 expression in HCT116 cells supplemented with different doses of NaCr for 48 h. ChIP-PCR assay analyzing the enrichment of GCN5 (**H**), H3K27cr (**I**), and YEATS2 (**J**) on ETS1 promoter. **K** qRT-PCR assessing the expression of ETS1 in HCT116 cells following co-transfection of LINC00887 plasmid with siGCN5 or siYEATS2. Data are represented as means ± SD, *P < 0.05, **P < 0.01, ***P < 0.001, ****P < 0.0001, unpaired, two-tailed, Student’s *t* test.

ETS1 regulates expression of genes of CAMs. We next investigated whether LINC00887 influenced ETS1 expression. Our observations revealed that LINC00887 overexpression increased ETS1 level, whereas LINC00887 knockdown exhibited the opposite effect (Fig. 7A–D). Additionally, a rescue experiment was conducted by co-transfecting HCT116 cells with the LINC00887 plasmid and siETS1, revealing that ETS1 knockdown restored the levels of invasion and migration, along with the expression of E-cadherin, N-cadherin, and vimentin (Fig. 7E, F), suggesting that LINC00887 regulated metastasis via ETS1.

Next, the regulatory mechanism of LINC00887 on ETS1 was studied. We found that ETS1 expression was dependent on NaCr concentration, with increasing NaCr concentration leading to increased ETS1 expression (Fig. 7G). Consequently, analysis of the ETS1 promoter revealed that LINC00887 overexpression increased the enrichment of GCN5, YEATS2 and H3K27cr in this region. Conversely, the LINC00887 knockdown had the opposite effect (Fig. 7H–J). A rescue experiment showed that GCN5 or YEATS2 knockdown restored the expression of ETS1 regulated by LINC00887 (Fig. 7K and Supplementary Fig. S6). Overall, the results indicated that LINC00887 enhanced the enrichment of GCN5, H3K27cr, and YEATS2 in the ETS1 promoter, activating ETS1 expression.

### Inhibition of LINC00887 suppresses CRC metastasis in vivo

**Fig. 8:**
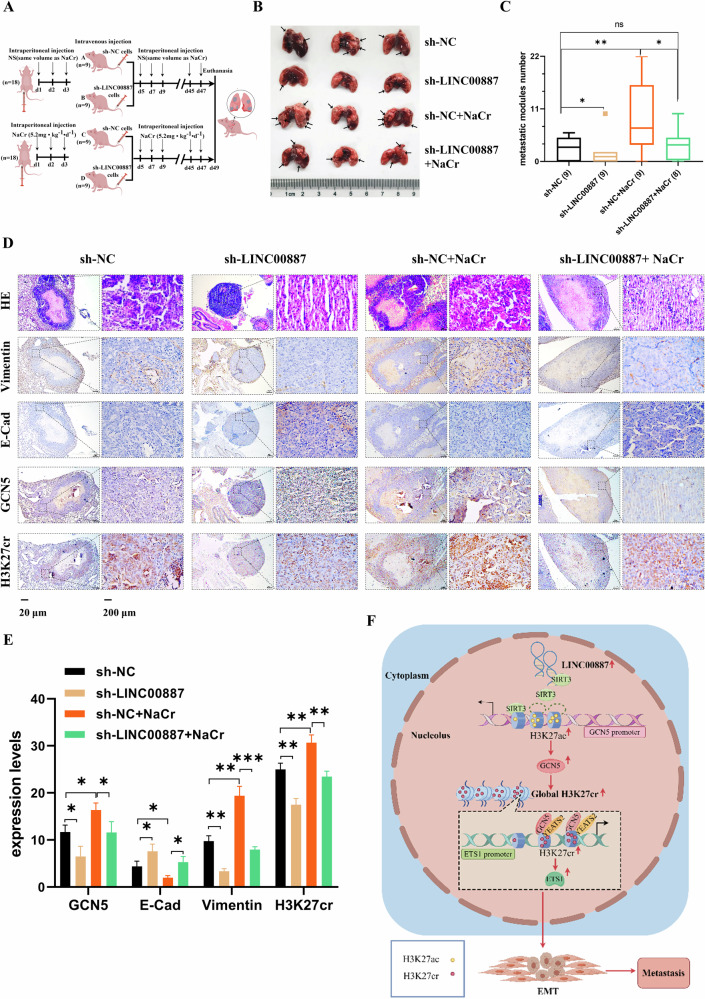
Inhibition of LINC00887 suppresses CRC metastasis in vivo. **A** Schematic diagram of the animal experiment. NS: normal saline. **B** Representative images showing metastatic nodules in the lung by LINC00887 stable cells in mice treated with or without NaCr. The arrows represent the tumor nodules. **C** Analysis of the number of metastatic nodules in the lungs using unpaired, one-tailed Student’s *t* test. The outlier in the sh-LINC00887 group was removed. One mouse in group D died on the second day after cell injection. **D** Representative images showing the levels of GCN5, H3K27cr, E-cadherin, and vimentin in metastatic nodules. Each group of slices comes from the same tumor tissue, although the directions of slices in sh-LINC00887 treated with NaCr and sh-NC groups are different. **E** The statistical results of IHC assay. The values were calculated in three random fields, finished by image J. **F** Model depicting the mechanism of LINC00887 in promoting CRC metastasis through H3K27cr.

Finally, we investigated the role of LINC00887 in regulating CRC metastasis in vivo (Fig. 8A). The results found that the LINC00887 suppression effectively inhibited the metastasis of HCT116 cells. However, this inhibitory effect was abolished when mice were treated with NaCr (Fig. 8B, C). Moreover, it was observed that inhibiting LINC00887 reduced GCN5, H3K27cr, and vimentin levels. These reductions were reversed by treatment with NaCr (Fig. 8D, E). In conclusion, our findings indicated that the interaction between LINC00887 and SIRT3 displaced SIRT3 from the GCN5 promoter. This displacement increased H3K27ac level at the GCN5 promoter, activating GCN5 expression and enhancing global level of H3K27cr. Consequently, recruitment of GCN5 to the ETS1 promoter was enhanced, elevating H3K27cr level in this region. This enhanced enrichment further promoted the recruitment of YEATS2, increasing ETS1 expression and CRC metastasis (Fig. 8F).

## Discussion

Elevated levels of lysine crotonylation (Kcr) have been observed in CRC tissues [27, 28], yet the underlying reasons for this upregulation and its impact on CRC patients remain incompletely understood. This study aimed to explore the mechanism responsible for H3K27cr elevation and its role in regulating CRC progression. Pervious research has indicated that lncRNAs are involved in regulating histone modification [29]. Consequently, we examined the correlation between lncRNAs and H3K27cr. Our findings suggested that LINC00887 was positively associated with the level of H3K27cr and genes with promoters occupied by H3K27cr. Our subsequent investigation focused on the molecular mechanism through which LINC00887 regulated H3K27cr. The results revealed that LINC00887 augmented the expression of GCN5, a crotonyltransferase upregulated in CRC tissues. Therefore, we proposed a model in which “LINC00887 enhanced H3K27cr level by upregulating GCN5 expression” to elucidate high level of H3K27cr in CRC tissues. Previous studies suggested that Kcr is generally associated with gene transcription activities [30]. As such, we examined the pathways annotated by genes whose promoters are occupied by H3K27cr in order to explore the function of H3K27cr. The findings revealed that metastatic-related pathways were significantly enriched, suggesting a connection between H3K27cr and metastasis. Our subsequent investigation concentrated on understanding the molecular mechanism by which H3K27cr regulated metastasis. The findings revealed that LINC00887 enhanced H3K27cr enrichment on the ETS1 promoter, subsequently recognized by YEATS2, ultimately promoting ETS1 transcription. ETS1, a transcription factor, is essential effector proteins that influences invasiveness and metastatic potential [31]. Therefore, thess findings suggested that H3K27cr potentially facilitated CRC metastasis via the YEATS2/ETS1 pathway.

Currently, colonoscopy is the gold standard for CRC screening [32]. However, the invasive and costly nature of colonoscopy necessitates the development of innovative and non-invasive approaches. Histone modifications have emerged as potential biomarkers for patient diagnosis and prognosis. Specifically, histone modifications, such as H3K27me3, H4K20me3, and H3K9me3, have been identified as potential diagnostic biomarkers for CRC [33]. Previous research has shown variations in pan-Kcr levels across multiple carcinoma types [28], suggesting the potential diagnostic value of lysine crotonylation biomarkers. Our research findings provide evidence for the diagnostic efficacy of H3K27cr in distinguishing CRC tissues from healthy controls. Further investigation is required to determine the efficacy of detecting H3K27cr level in blood or stool samples to distinguish patients with CRC from healthy individuals. Furthermore, various histone modifications, such as H3K9me3, H3K20me3, H4K16ac, and H3K56ac, have been identified as potential prognostic biomarkers in CRC [33]. Our research findings suggested that elevated level of H3K27cr were linked to unfavorable survival outcomes in CRC patients with lymph node invasion, underscoring the significance of H3K27cr as a prognostic marker. It is imperative to conduct further analyses using larger clinical samples to validate these findings. Exploring the diagnostic and prognostic implications of histone crotonylation is likely to emerge as a future research trend, encompassing identifying specific sites.

Our study demonstrated that mice supplemented with NaCr exhibited increased metastatic nodules in the lungs, indicating that elevated crotonylation level could promote CRC metastasis. These findings suggest that cancer patients might delay tumor metastasis by restricting their dietary consumption of foods that could generate crotonyl-CoA because cancer cells undergo metabolic reprogramming to support tumorigenicity and malignancy [34]. Lysine crotonylation requires a crotonyl donor derived from crotonyl-CoA. Crotonyl-CoA can be obtained from various sources, including lysine catabolism [30], fatty acid β-oxidation [35], gut microbiome-derived short-chain fatty acids (SCFAs) [36], and exogenous supplementation with crotonic acid [37]. Consequently, a diet restricted to lysine can improve the survival rate of mice [30]. Moreover, research has proved that cancer cells exhibit an elevated fatty acid β-oxidation level [38, 39], possibly leading to heightened production of crotonyl-CoA and subsequent evaluation of crotonylation level, ultimately promoting cancer metastasis. Our findings further suggested that therapeutic interventions targeting crotonylation could potentially serve as an innovative approach in the fight against cancer. Consequently, future efforts in drug development may require research on drugs that target crotonylation.

Previous research has indicated that GCN5 functions as an acetyltransferase and is a catalytic subunit of the histone acetyltransferase (HAT) module within Ada-two-A-containing (ATAC) complexes [40]. These complexes serve as transcriptional co-activators and regulate chromatin accessibility [41, 42]. YEATS2 acts as a subunit and selective histone acetylation reader within the ATAC complex [43]. YEATS2 also functions as a histone crotonylation reader, with a preference for H3K27cr over other histone acylations [16]. Our study showed that LINC00887 facilitates the accumulation of GCN5, H3K27cr, and YEATS2 in ETS1 promoter, further validating the involvement of histone crotonylation in the recruitment of ATAC complexes.

## Supplementary information

Supplementary Materials

Supplementary Table S3

Raw data for Western Blot Experiments

## Data Availability

The ChIP-seq data of H3K27cr (GSE128590) is available from the corresponding author upon reasonable request. The information of COAD tissues from the TCGA dataset is available in the UCSC Xena TCGA hub repository (https://xenabrowser.net/hub/). The protein level of GCN5 is available in the human protein atlas (HPA) database (https://www.proteinatlas.org/). The datasets of GSE15960, GSE110009, GSE163974, GSE221575, GSE225857 are available in the GEO database (https://www.ncbi.nlm.nih.gov/gds/). The gene set of EMT pathway is available in the MSigDB database (https://www.gsea-msigdb.org/gsea/msigdb/index.jsp).
