# Supplementary material for: LINC00887 promotes GCN5-dependent H3K27cr level and CRC metastasis via recruitment of YEATS2 and enhancing ETS1 expression
Source: Cell Death Dis. 2024 Sep 30;15(9):711. doi: 10.1038/s41419-024-07091-w (PMC11443008; doi:10.1038/s41419-024-07091-w)
Supplement: Supplementary file 1 — Supplementary Materials [file 41419_2024_7091_MOESM1_ESM.docx]

**Supplementary Materials**

**LINC00887 promotes GCN5-dependent H3K27cr levels and CRC metastasis via recruitment of YEATS2 and enhancing ETS1 expression**

Meijian Liao^1^, Wendan Zheng^1^, Yifan Wang^1^, Mengting Li^1^, Xiaolin Sun^1^, Nan Liu^2,3^, Jia Yao^1^, Fuxing Dong^4^, Qingling Wang^1^, Yu Ma^1*^, Jie Mou^5*^

^1^Department of Pathology, Xuzhou Medical University, Xuzhou 221004, P.R. China

^2^ Department of Infectious Diseases and Center of Infectious Diseases and Pathogen Biology, The First Hospital of Jilin University, Changchun 130061, P.R. China

^3^Key Laboratory of Organ Regeneration and Transplantation of the Ministry of Education, The First Hospital of Jilin University, Changchun 130061, P.R. China

^4^Public Experimental Research Center, Xuzhou Medical University, Xuzhou 221004, P.R. China

^5^School of Pharmacy, Xuzhou Medical University, Xuzhou 221004, P.R. China

Meijian Liao and Wendan Zheng contribute to this work equally.

**Running title:** LINC00887 facilitates CRC metastasis via H3K27cr

**Conflict of interest:** The authors declare no potential conflicts of interest

**Keywords:** Colorectal cancer, histone crotonylation, lncRNA, LINC00887, H3K27cr, GCN5

***Correspondence:**

mou.jie@xzhmu.edu.cn (Jie Mou)

mayuin1107@163.com (Yu Ma)

**
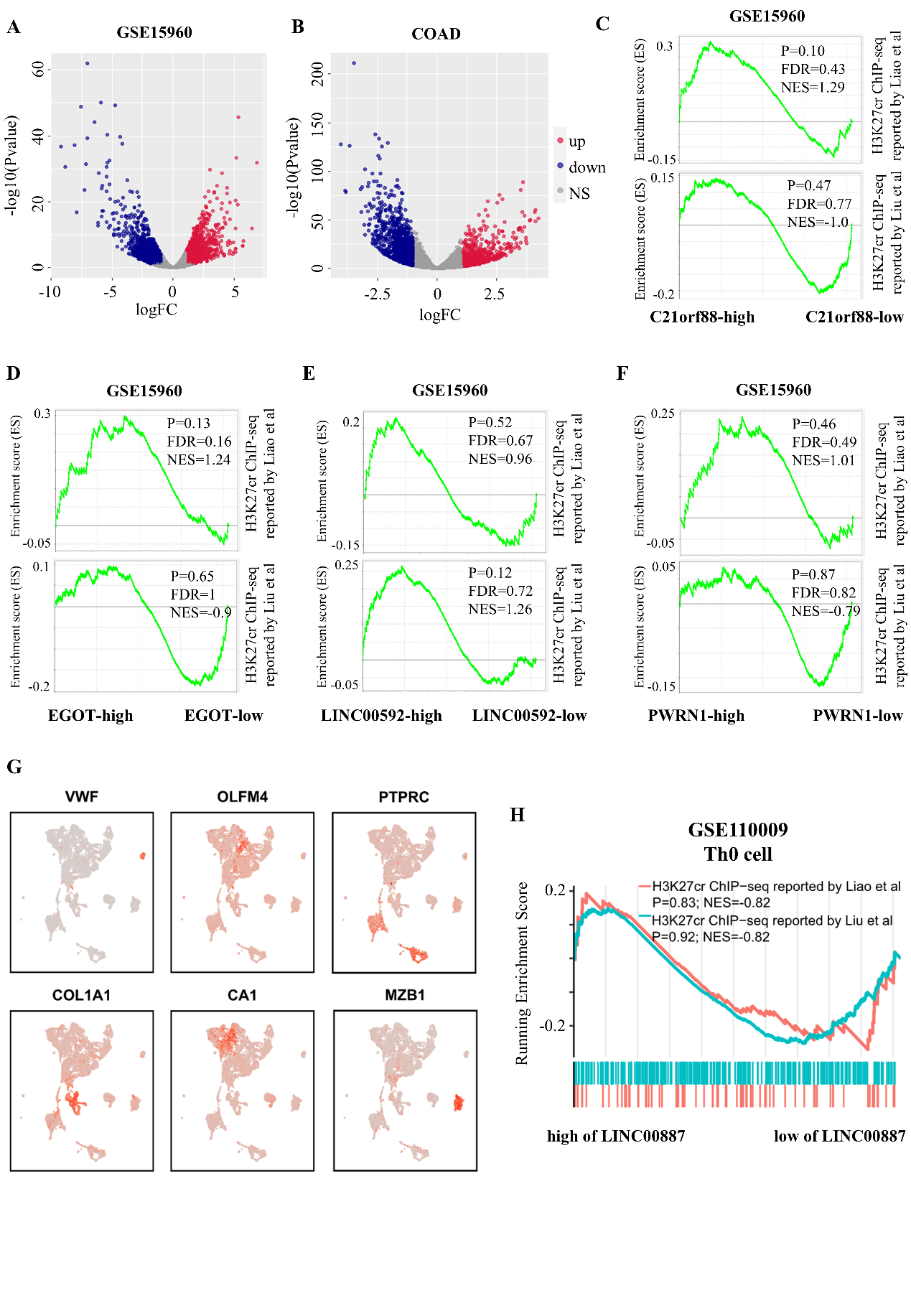
**

**Fig. S1. The association between lncRNAs and H3K27cr levels.** **A** Volcano showing genes differently expressed in epithelial cells derived from normal and CRC patients (GSE15960). Each point indicates one gene. The blue and red indicate genes that are down-regulated and up-regulated in epithelial cells derived from CRC tissues, respectively. **B** Volcano showing genes differently expressed between normal and COAD tissues from the TCGA database. Each point indicates one gene. The blue and red indicate genes that are down-regulated and up-regulated in COAD tissues, respectively. **C-F** The analysis of genes with promoters occupied by H3K27cr in epithelial cells derived from CRC tissues (GSE15960) with high or low expression of C21orf88 (**C**), EGOT (**D**), LINC00592 (**E**), and PWRN1 (**F**). **G** Scatter plotting the expression of marker genes across different types of cells from CRC tissues (GSE110009). **H** Fgsea showing the enrichment of genes with promoters occupied by H3K27cr in Th0 cells with high or low expression of LINC00887.

**
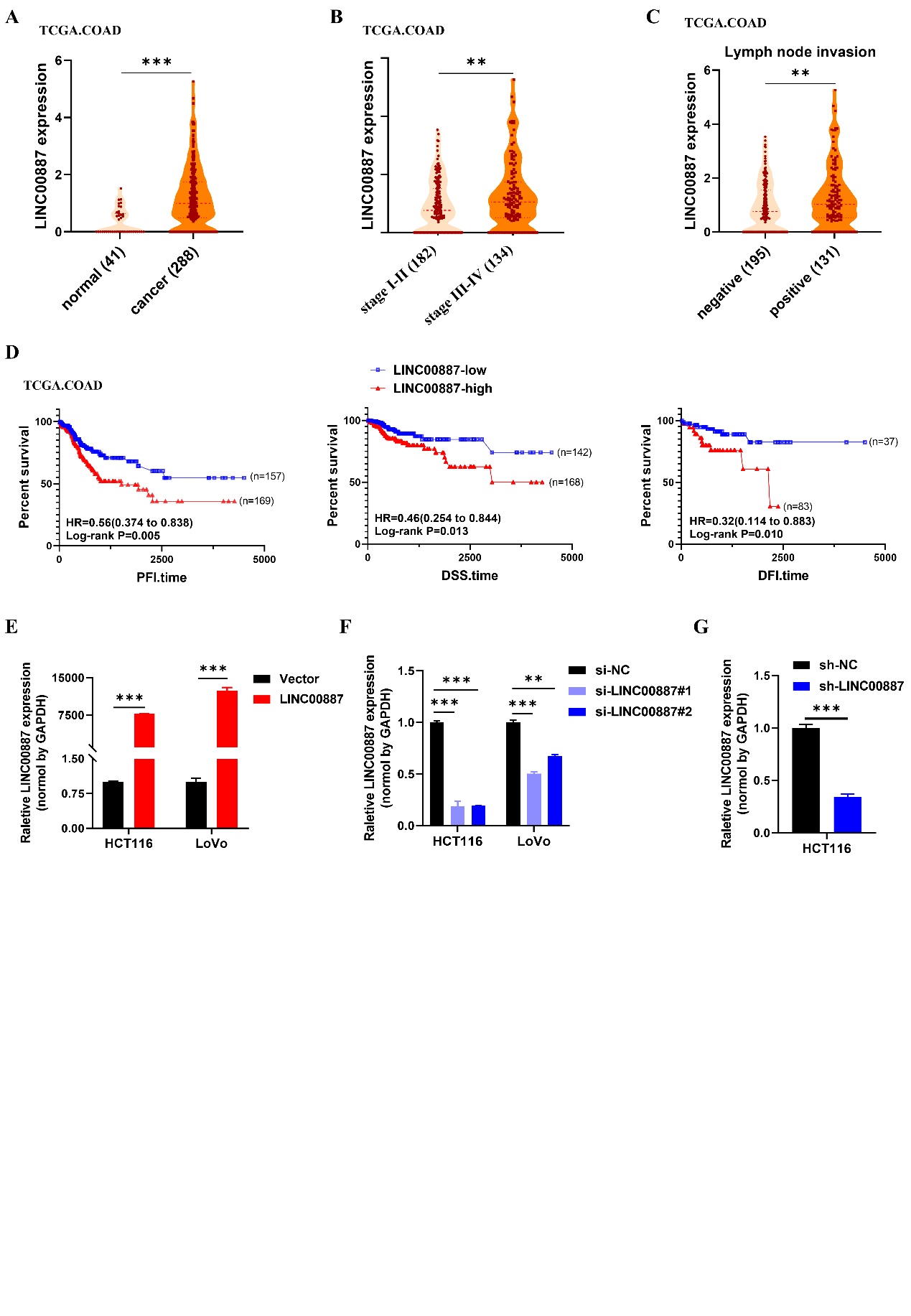
**

**Fig. S2. The association between LINC00887 and clinicopathological characteristics.** **A.** Analysis of LINC00887 expression between normal and COAD tissues from the TCGA dataset. **B-C** Analysis of LINC00887 expression in COAD tissues with low and high stage (**B**), or negatively and positively of lymph node invasion (**C**). **D** Kaplan-Meier survival curve analysis of the association of LINC00887 expression with progression-free interval (PFI, left panel), disease-specific survival (DSS, middle panel), disease-free interval (DFI, right panel) in COAD patients. The cut off values were determined according the maximum value of Yoden index, analyzed by ROC curve. **E-G** The qRT-PCR assay analysis of LINC00887 expression in CRC cells transfected with LINC00887 plasmid (**E**) or siLINC00887 (**F**) for 48 h, or in HCT116 cells with LINC00887 stable knockdown (**G**). Data are represented as means ± SD, ***P* < 0.01, ****P* < 0.001, unpaired, two-tailed, Student's *t*-test.

**
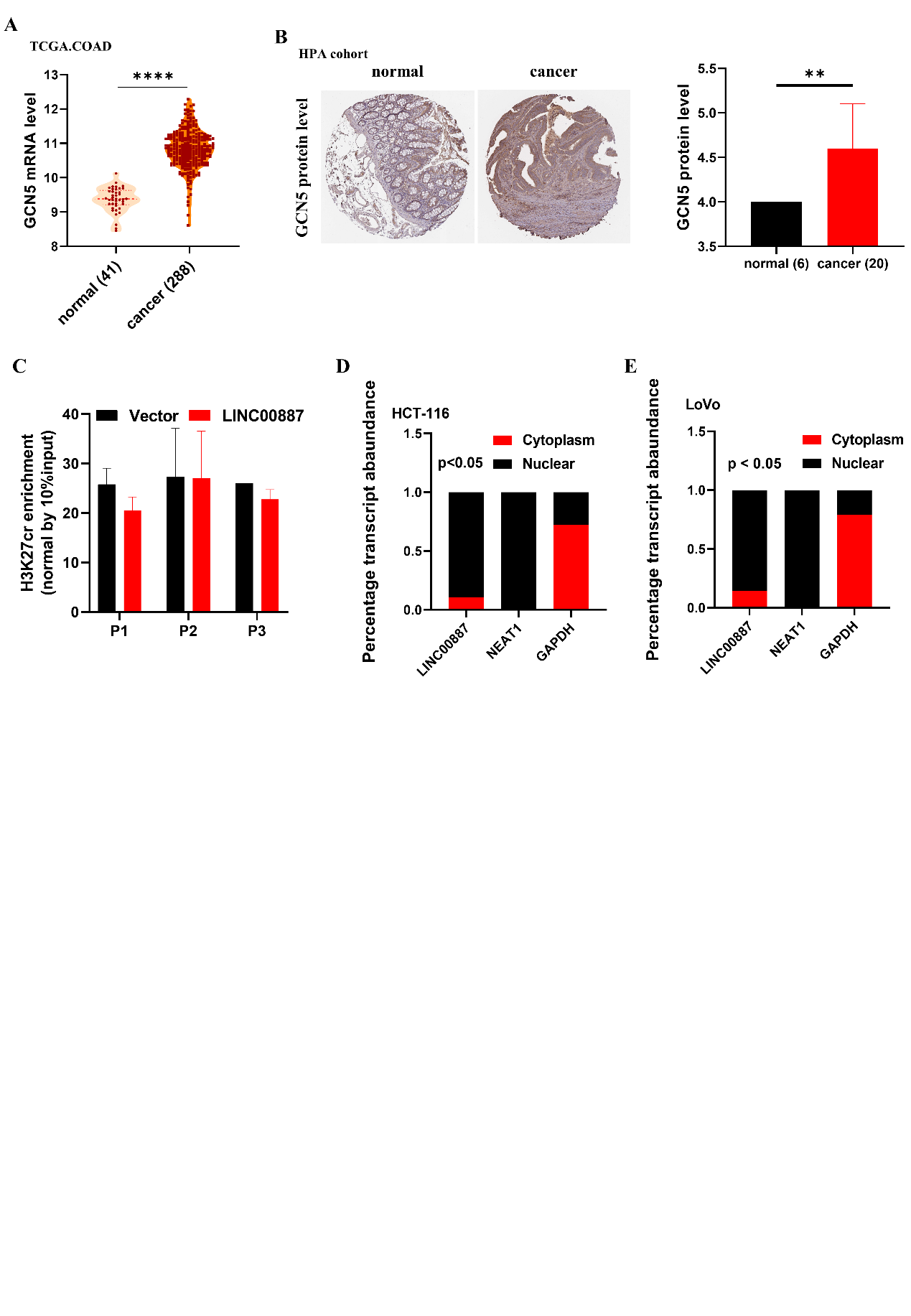
**

**Fig. S3. The distribution of LINC00887.** **A** Mann-Whitney U analysis of GCN5 level between normal and CRC tissues from the TCGA cohort. **B** Representative images from the Human protein atlas (HPA) database (left panel) and Student's *t*-test showing GCN5 levels between normal and CRC tissues (right panel). **C** The ChIP-PCR assay analysis of the enrichment of H3K27cr in GCN5 promoter in HCT116 cells with instantaneous overexpression of LINC00887. **D-E** Analysis of the distribution of LINC00887 between nucleus and cytoplasm in HCT116 (**D**) and LoVo (**E**) cells. NEAT1 and GAPDH act as controls. Data are represented as means ± SD, ***P* < 0.01, *****P* < 0.0001, unpaired, two-tailed, Student's *t*-test.

**
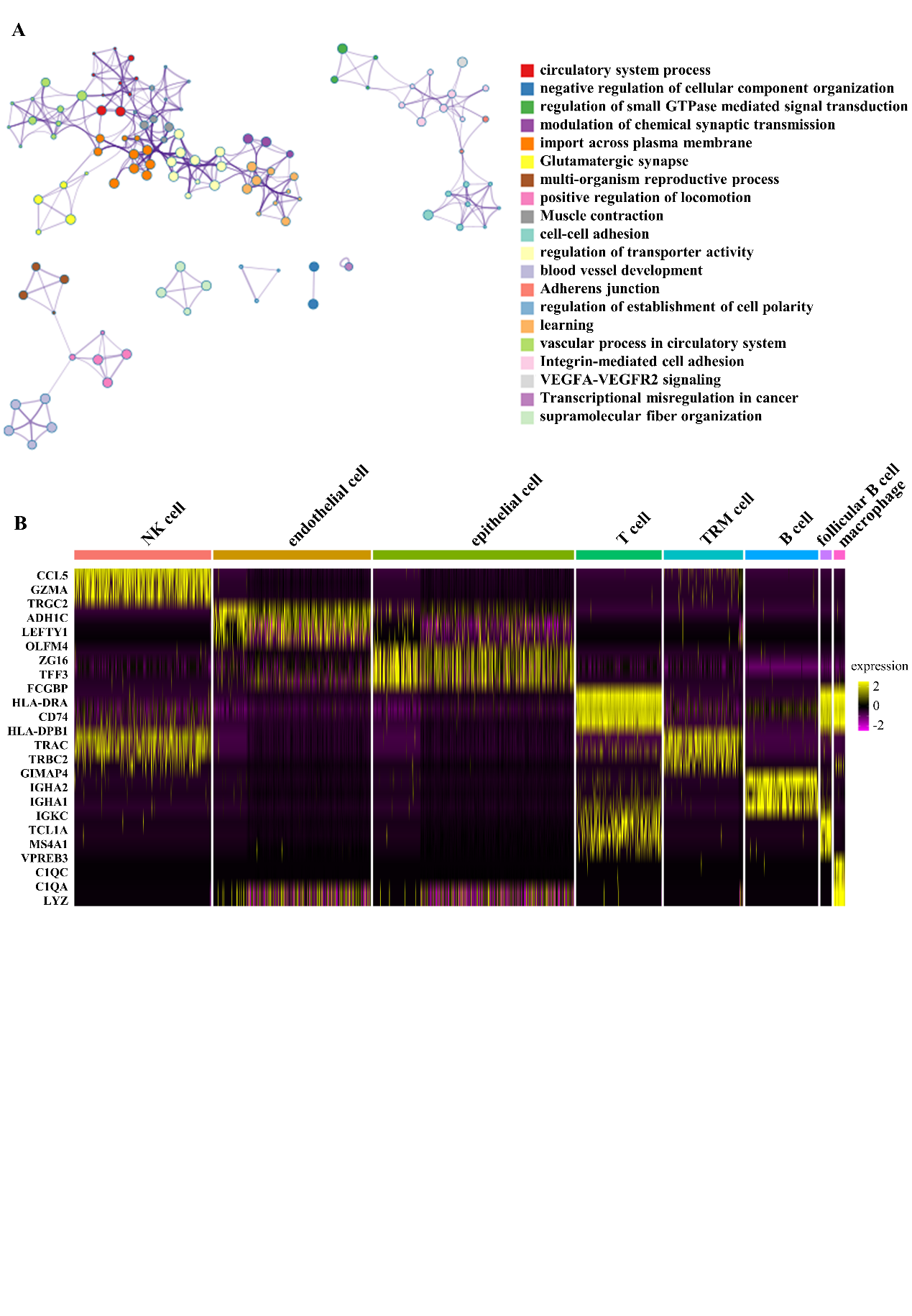
**

**Fig. S4. Single-cell transcriptome profiles.** **A** Over-representation analysis (ORA) of 739 genes using Metascape platform. The node represents an enriched term. The size of node represents the number of enriched genes, and color of node represents functional terms. **B** Heatmap displaying levels of marker genes in different cell types of CRC tissues (GSE163974).

**
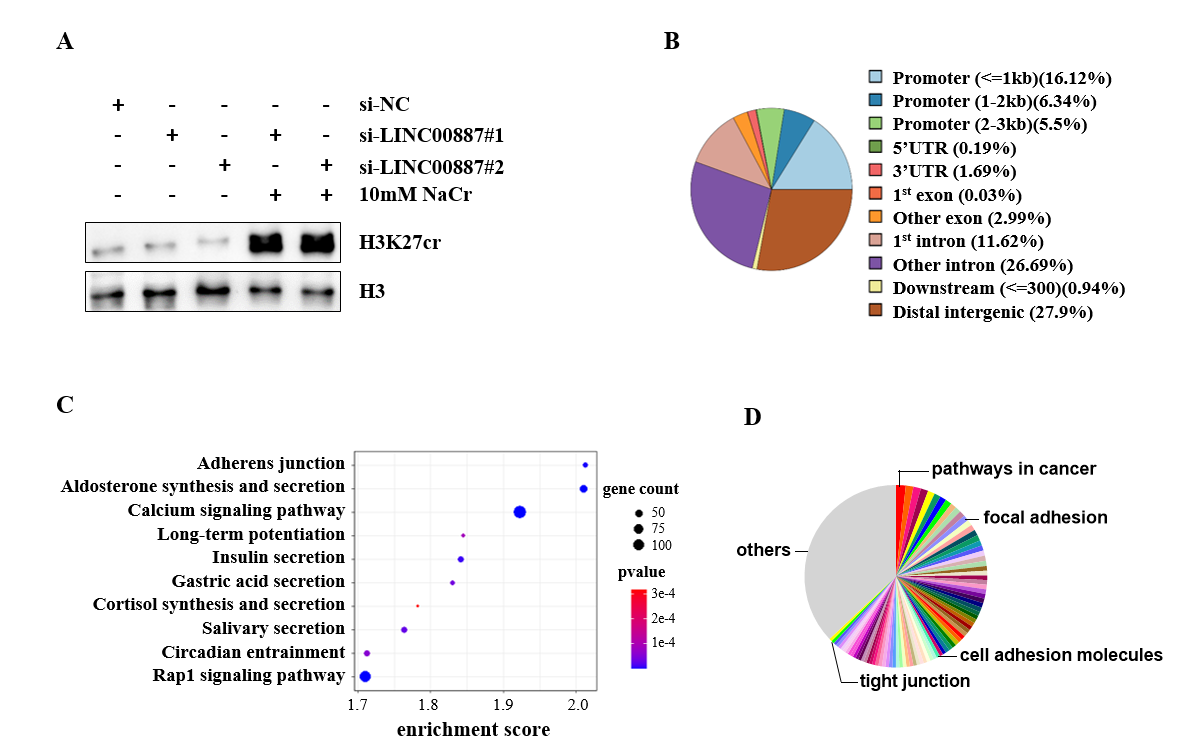
**

**Fig. S5. Characteristics of 602 genes.** **A** Immunoblotting analysis of H3K27cr level in HCT116 cells transfected with siLINC00887 and supplemented with NaCr for 48 h. The H3 acts as a loading control. **B** The location of 17,740 differential peaks on chromosomes. **C** KEGG analyzing the enrichment of biological pathways of genes annotated by above 17,740 differential peaks. **D** The functional pathways annotated by 602 genes.

**
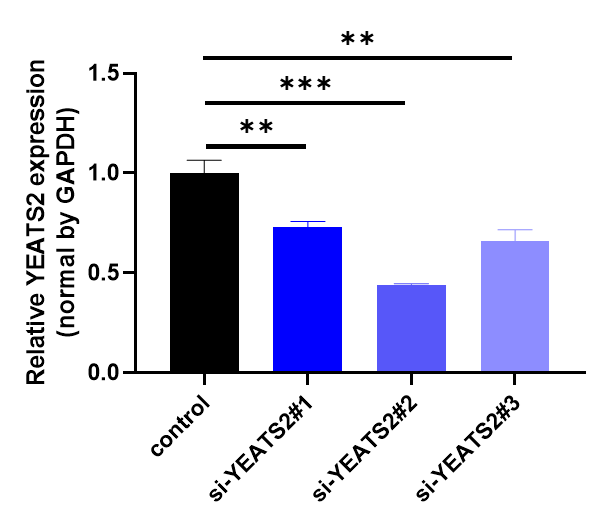
**

**Fig. S6.** The qRT-PCR assay analysis of YEATS2 expression in HCT116 cells transfected with siYEATS2 for 48 h. Data are represented as means ± SD, ***P* < 0.01, ****P* < 0.001, unpaired, two-tailed, Student's *t*-test.

**Supplementary Table S1.** The sequences of siRNAs and shRNA.

| genes | Sequence (5’-3’) |
| --- | --- |
| si-LINC00887#1 sh-LINC00887 | CAAUGAAACAGAACAGAUATT |
| si-LINC00887#2 | AUCAGUAAGUGUUUGCUAATT |
| si-GCN5 | CAAUGAAACCUGUAAGUGUTT |
| si-ETS1 | ACUUGCUACCAUCCCGUACTT |
| si-YEATS2#1 | GGAUAGAUAUCAUACAUAAUCTT |
| si-YEATS2#2 | GCAGUAUGUGACUGUGAAAGGTT |
| si-YEATS2#3 | AGAACAGUUUGCUCUUGAATTTT |

**Supplementary Table S2.** The sequences of primers used in this study.

|  | Forward primer (5’-3’) | Reverse primer (5’-3’) |
| --- | --- | --- |
| LINC00887 | TCCTCCTTCTCCATCCTTGCTCTG | CCTGCTCCCAGTCACCATTGAATC |
| ETS1 | TTGAAAGCATAGAGAGCTACGA | CTCTGAGTCGAAGCTGTCATAG |
| GCN5 | CAGTTTCGGCAGAGGTCTCA | ATGAGTGGTTTCGTAGCGGG |
| GAPDH | GAAGGTGAAGGTCGGAGTC | GAAGATGGTGATGGGATTTC |
| GCN5-P1 | GGTTGGAAGCCTTAGAGCC | CAGTTACCGCATGTCACAGAA |
| GCN5-P2 | GCTCGGTTCCCTTCGCTTCT | CGCTGACAGTCTGGCTCTTG |
| GCN5-P3 | GACACGAGGAGGTGGACTGG | CAAGAAGGCTACTGACGTGGAG |
| ETS1-P1 | CCTCCTTCAGAAACACACGC | CGTGAGGCATGTGGATGAAG |
| ETS1-P2 | CTTCATCCACATGCCTCACG | AGTCCGTCTGATTCTCCACG |
| ETS1-P3 | TGGAGGCCAGCATTGTTTTC | GGCTCCTGGTCTAAACTCCA |
| ETS1-P4 | GGAGTTTAGACCAGGAGCCA | GCACATTTGAACTCCCAGCA |

**The R code of scRNA-seq data processing**

library(Seurat)

library(presto)

library(dplyr)

library(msigdbr)

library(tibble)

library(fgsea)

library(ggplot2)

# Building seurat object of samples from the GSE163974 datasets#

seurat1 <- readRDS('GSM4994385.rds')

seurat2 <- readRDS('GSM4994386.rds')

pdf("seurat1-QC.pdf",width=11)

VlnPlot(seurat1, features = c("nFeature_RNA", "nCount_RNA"), ncol = 3)

dev.off()

pdf("seurat2-QC.pdf",width=11)

VlnPlot(seurat2, features = c("nFeature_RNA", "nCount_RNA"), ncol = 3)

dev.off()

mito.genes <- grep(pattern = "^MT-",

x = rownames(seurat1@assays[["RNA"]]),

value = TRUE)

seurat1[["percent.mt"]] <- PercentageFeatureSet(seurat1, pattern = "^MT-")

seurat1 <- subset(seurat1, subset = nFeature_RNA > 200 & nFeature_RNA < 1000 & percent.mt < 20)

mito.genes <- grep(pattern = "^MT-",

x = rownames(seurat2@assays[["RNA"]]),

value = TRUE)

seurat2[["percent.mt"]] <- PercentageFeatureSet(seurat2, pattern = "^MT-")

seurat2 <- subset(seurat2, subset = nFeature_RNA > 200 & nFeature_RNA < 3000 & percent.mt <20)

seurat <- FindIntegrationAnchors(object.list = list(seurat1, seurat2), dims = 1:30)

seurat <- IntegrateData(anchorset = seurat, dims = 1:30)

seurat <- SCTransform(seurat)

seurat <- RunPCA(seurat, features = VariableFeatures(object =seurat))

pdf("Elbow.pdf")

ElbowPlot(seurat,ndims = 30)

dev.off()

# Building seurat object of samples from the GSE110009 datasets#

seurat_data<- read.table("GSE110009_metastatic_Colon_TPM.txt", row.names = 1, header = TRUE, sep = "\t")

seurat_obj <- CreateSeuratObject(counts = seurat_data,

min.features = 200,

min.cells = 3,

project = "GSE110009")

scRNA <- NormalizeData(seurat_obj, normalization.method = "LogNormalize", scale.factor = 10000)

scRNA <- FindVariableFeatures(scRNA, selection.method = "vst", nfeatures = 2000)

all.genes <- rownames(scRNA)

scRNA <- ScaleData(scRNA, features = all.genes)

scRNA <- RunPCA(scRNA, features = VariableFeatures(object = scRNA))

pdf("Elbow.pdf")

ElbowPlot(scRNA,ndims = 50)

dev.off()

# Integration between samples to remove the batch effect#

scRNA.list <- SplitObject(scRNA, split.by = "orig.ident")

scRNA.list <- lapply(X = scRNA.list, FUN = function(x) {

x <- NormalizeData(x, verbose = FALSE)

x <- FindVariableFeatures(x, verbose = FALSE)

})

features <- SelectIntegrationFeatures(object.list = scRNA.list)

anchors <- FindIntegrationAnchors(object.list = scRNA.list, reduction = "rpca",dims = 1:50,k.filter = 100)

scRNA.integrated <- IntegrateData(anchorset = anchors, dims = 1:30)

scRNA.integrated <- ScaleData(scRNA.integrated, verbose = FALSE)

scRNA.integrated <- RunPCA(scRNA.integrated, verbose = FALSE)

pdf("ElbowIntegrated.pdf")

ElbowPlot(scRNA.integrated,ndims = 30)

dev.off()

scRNA.integrated<- FindNeighbors(scRNA.integrated, reduction = "pca", dims = 1:30)

DefaultAssay(scRNA.integrated)="integrated"

scRNA.integrated<- FindClusters(scRNA.integrated, resolution = 1.5)

scRNA.integrated<- RunUMAP(scRNA.integrated, reduction = "pca", dims = 1:30)

pdf("scRNAIntegratedSample.pdf",width=9)

DimPlot(scRNA.integrated,reduction="umap",group.by="orig.ident")

dev.off()

pdf("scRNAIntegratedCluster.pdf",width=9)

DimPlot(scRNA.integrated,reduction="umap",label=TRUE)

dev.off()

#identification the cell type associated markers#

scRNA.integrated.cellType=subset(scRNA.integrated,idents=c(0:12)) #The parameters for GSE163974 dataset#

scRNA.integrated.cellType=subset(scRNA.integrated,idents=c(0:29)) #The parameters for GSE110009 dataset#

scRNA.integrated.celltype.markers <- FindAllMarkers(scRNA.integrated.cellType, only.pos = TRUE, min.pct = 0.25, logfc.threshold = 0.25)

write.table(scRNA.integrated.celltype.markers,file="scRNA.integrated.cellType.markers.txt",sep="\t",quote=F)

scRNA.integrated.celltype.markers %>%

group_by(cluster) %>%

top_n(n = 3, wt = avg_log2FC) -> top3

scRNA.integrated.cellType <- ScaleData(scRNA.integrated.cellType, verbose = FALSE)

pdf("cellTypeMarkerDoHeatmap.pdf",width=20,height=15)

DoHeatmap(scRNA.integrated.cellType, features = top3$gene)

dev.off()

#identification the cell type using CellMarker 2.0 database and following code#

new.cluster.ids <- c("NK cell", "endothelial cell", "epithelial cell", "T cell", "TRM cell", "B cell", "endothelial cell", "epithelial cell", "epithelial cell", "epithelial cell", "NK cell", "follicular B cell", "macrophage") #The parameters for GSE163974 dataset#

new.cluster.ids <- c("enterocyte", "mesenchymal cell", "epithelial", "fibroblast", "enterocyte", "enterocyte", "enterocyte", "B cell", "neutrophil", "enterocyte", "goblet cell", "TRM cell", "mesenchymal cell", "fibroblast", "enterocyte", "cancer stem cell", "neutrophil", "cytotoxic T cell", "paneth cell", "epithelial", "enterocyte", "fibroblast", "endothelial cell", "neutrophil", "TRM cell", "fibroblast", "mast cell", "neutrophil", "Th0 cell", "enteroendocrine cell") #The parameters for GSE110009 dataset#

names(new.cluster.ids) <- levels(scRNA.integrated.cellType)

scRNA.integrated.cellType <- RenameIdents(scRNA.integrated.cellType, new.cluster.ids)

scRNA.integrated.cellType$cellType=Idents(scRNA.integrated.cellType)

pdf("scRNA.integrated.cellTypeType.Cluster2.pdf",width=9.5)

DimPlot(scRNA.integrated.cellType,reduction="umap",label=TRUE)&theme(panel.border = element_rect(fill=NA,color="black", size=1.5, linetype="solid"))

dev.off()

pdf("cellTypeMarkerDoHeatmap-celltype.pdf",width=20,height=15)

DoHeatmap(scRNA.integrated.cellType, features = top3$gene)

dev.off()

#fgsea analysis of the association #

scRNA.integratedMarker <- wilcoxauc(scRNA.integrated.cellType, 'cellType')

table(scRNA.integratedMarker$group)

for(cluster in unique(scRNA.integrated.cellType$cellType)){

print (cluster)

clusterCell<- scRNA.integratedMarker %>% dplyr::filter(group == cluster) %>% arrange(desc(logFC)) %>% dplyr::select(feature, logFC)

ranks<- deframe(clusterCell)

fgseaRes<- fgseaMultilevel(fgsea_sets, stats = ranks,eps=0, nPermSimple = 10000)

ranks=na.omit(ranks)

fwrite(fgseaRes, file=paste0("/",cluster,".txt",sep=""), sep="\t", sep2=c("", " ", ""))

}

#visualization result of fgsesa using scatter plot#

library(ggplot2)

data.final<-read.csv("GSEA.csv",header=T)

ggplot(data.final,aes(x=celltype,y=pathway,shape=factor(Sig)))+

geom_point(aes(size=`pval`,

color=`NES`))+

theme_bw()+

theme(panel.grid = element_blank(),

axis.text.x=element_text(angle=90,hjust = 1,vjust=0.5))+

scale_color_gradient(low="blue",high="red")+

labs(x=NULL,y=NULL)

#visualization result of fgsesa using heatmap#

library(gplots)

a <- read.csv('heatmap.csv', header = T, check.names = F, row.names = 1)

d <- as.matrix(a)

heatmap.2(d, Colv = F, Rowv = F, col=colorRampPalette(c("brown","white")),colsep=c(1:ncol(d)),rowsep=c(1:nrow(d)), sepcolor = "pink",symkey = F,trace="none")
