# Supplementary material for: LINC00887 promotes GCN5-dependent H3K27cr level and CRC metastasis via recruitment of YEATS2 and enhancing ETS1 expression
Source: Cell Death Dis. 2024 Sep 30;15(9):711. doi: 10.1038/s41419-024-07091-w (PMC11443008; doi:10.1038/s41419-024-07091-w)

Figure 2I

HCT116

H3K27cr

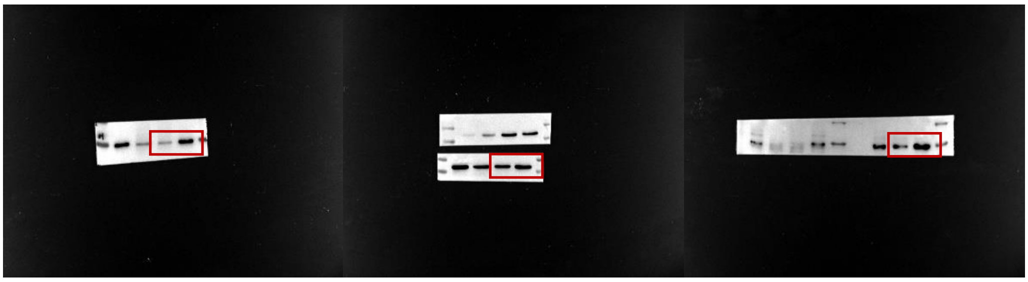

H3

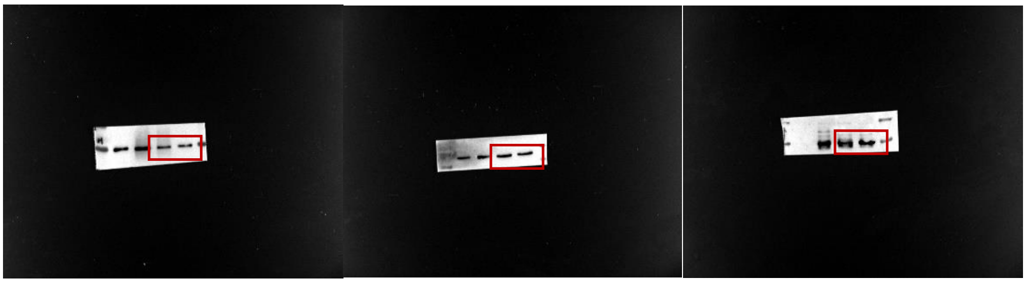

LOVO

H3K27cr

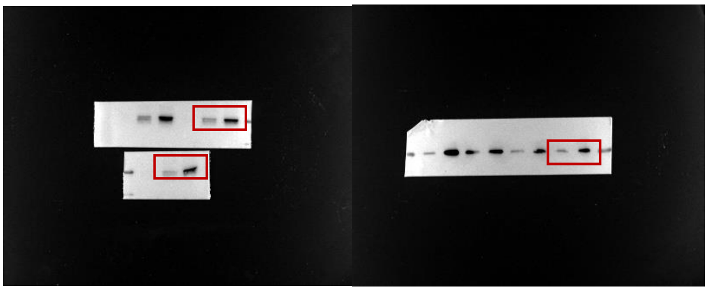

H3

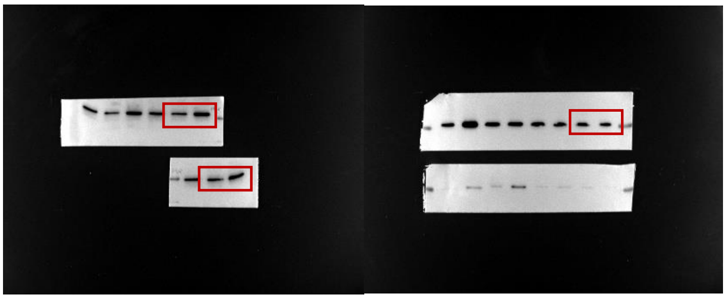

Figure 2J

HCT116

H3K27cr

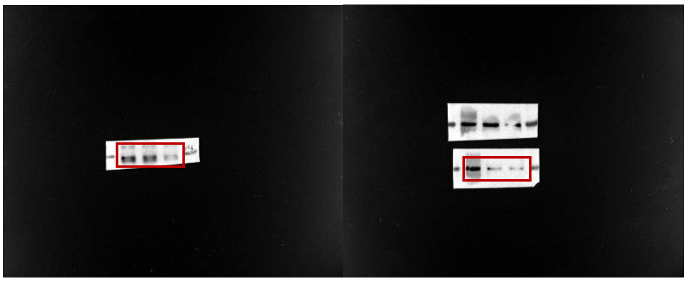

H3

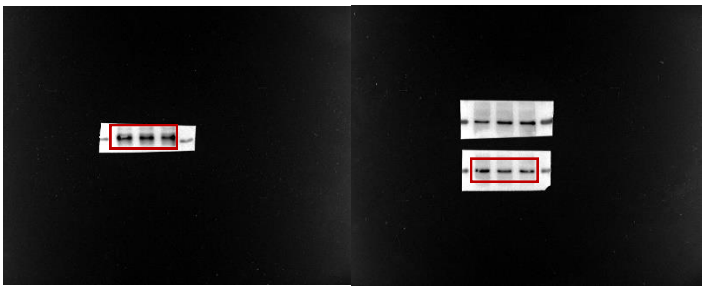

LOVO

H3k27cr

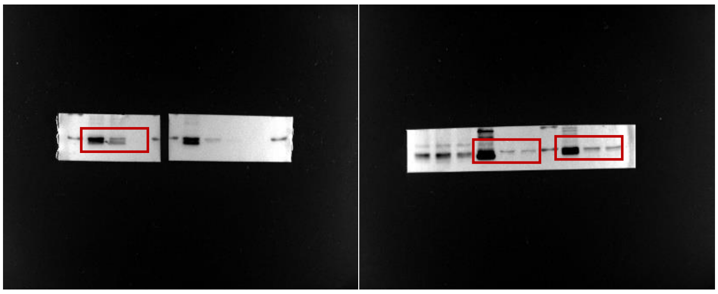

H3

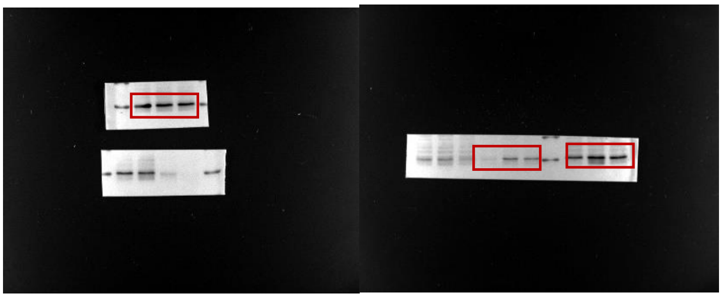

Figure 2K

H3k27cr

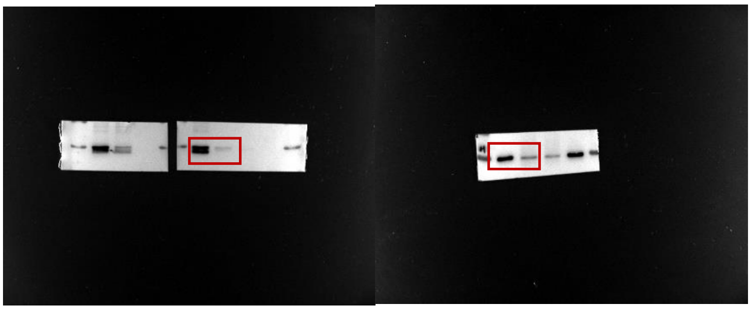

H3

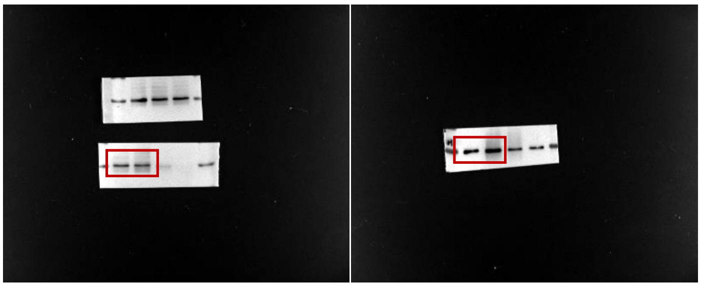

Figure 3B

GCN5

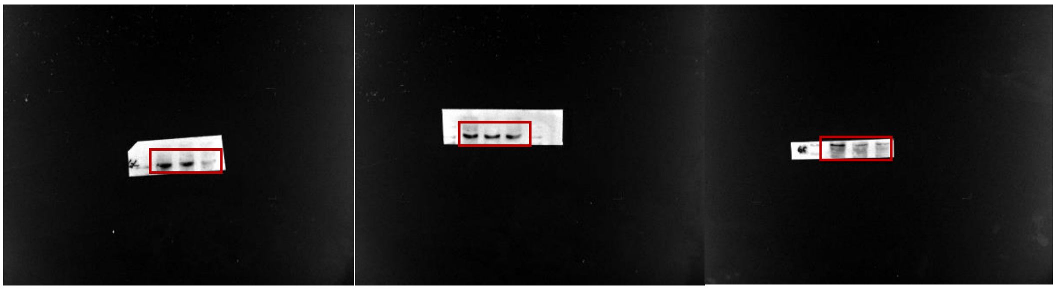

GAPDH

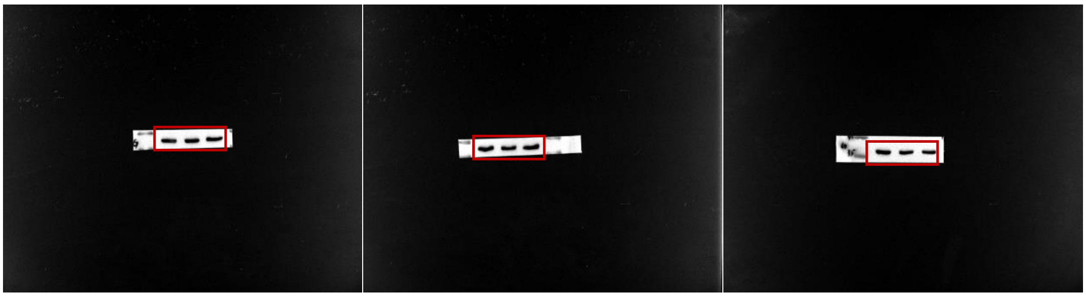

Figure 3C

GCN5

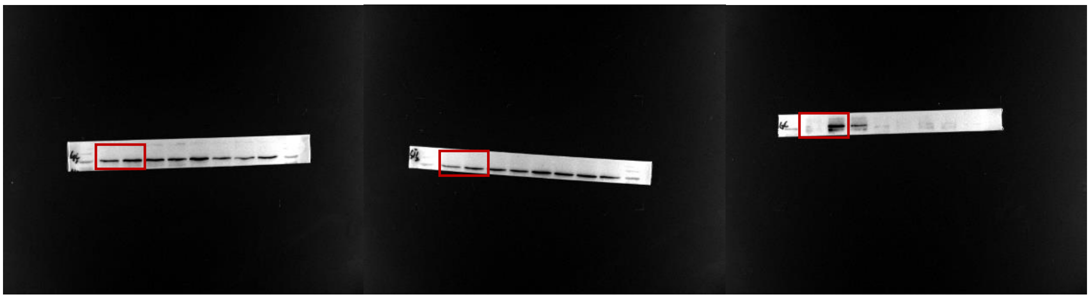

GAPDH

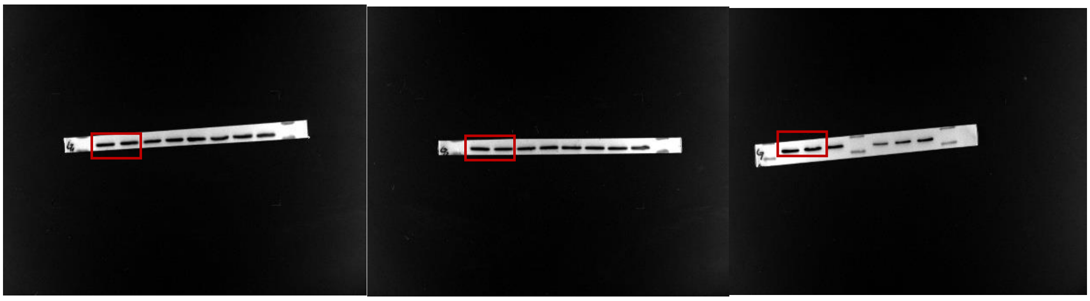

Figure 3D

GCN5

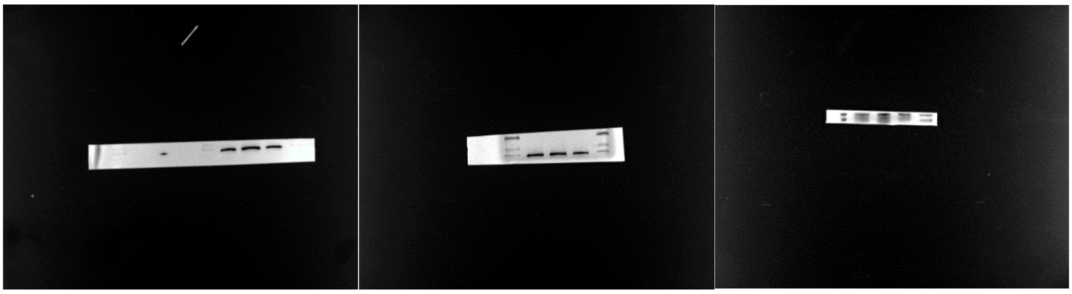

GAPDH

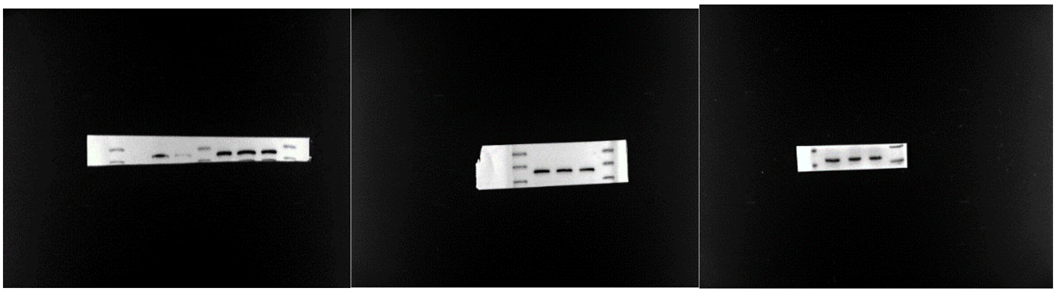

H3K27cr

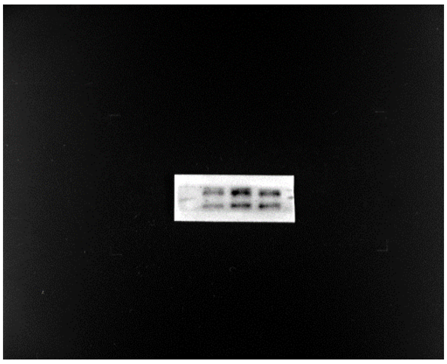

H3

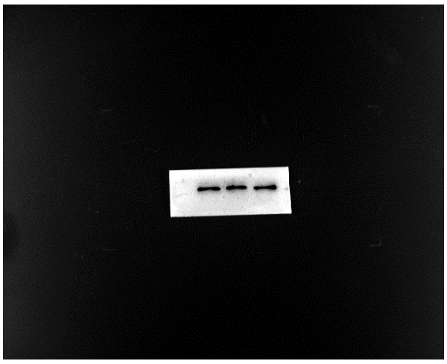

Figure 4F

E-cadherin

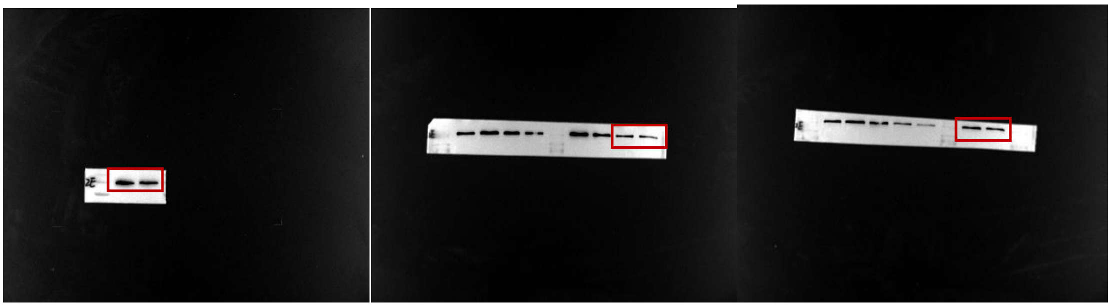

GADPH

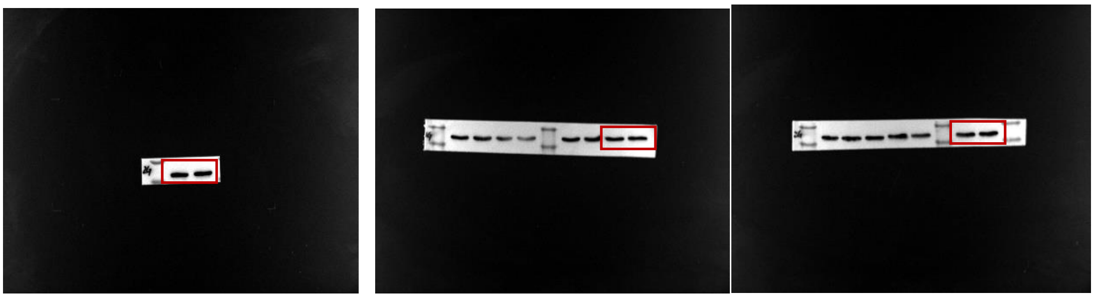

N-cadherin

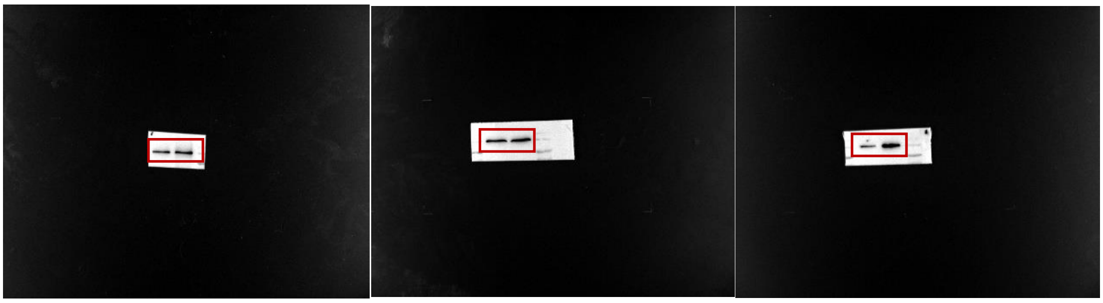

GAPDH

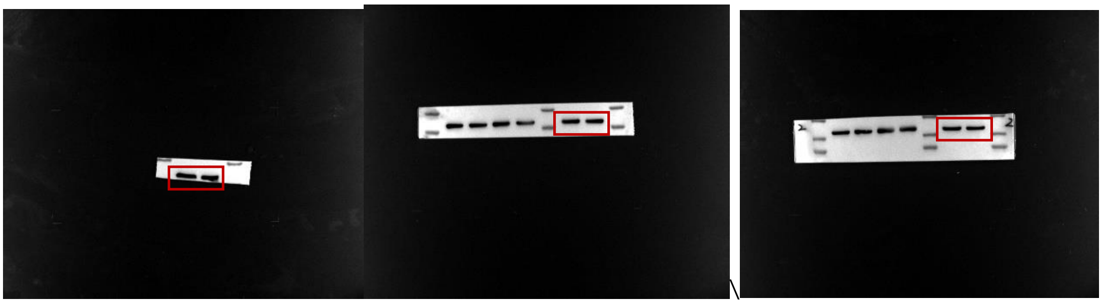

H3K27cr

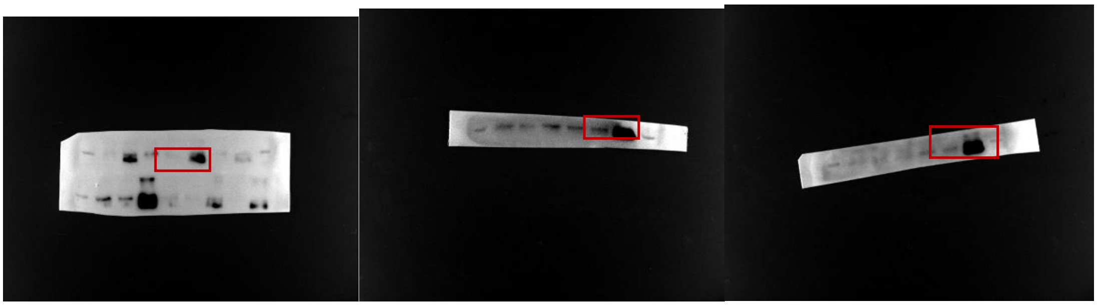

H3

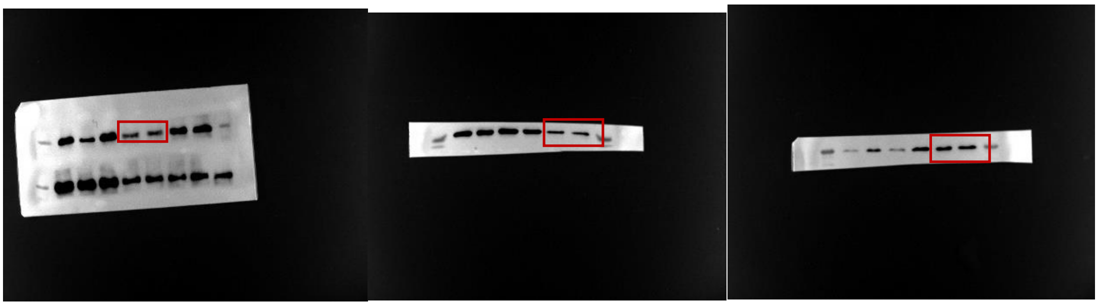

Figure 4G

E-cadherin

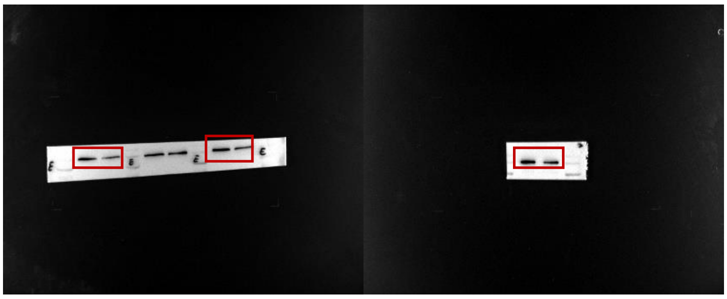

GAPDH

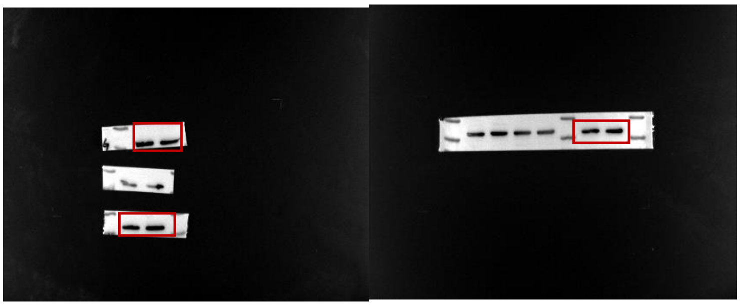

N-cadherin

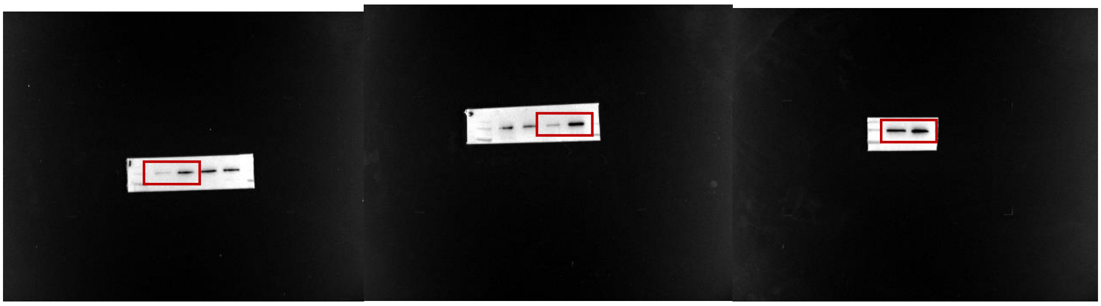

GAPDH

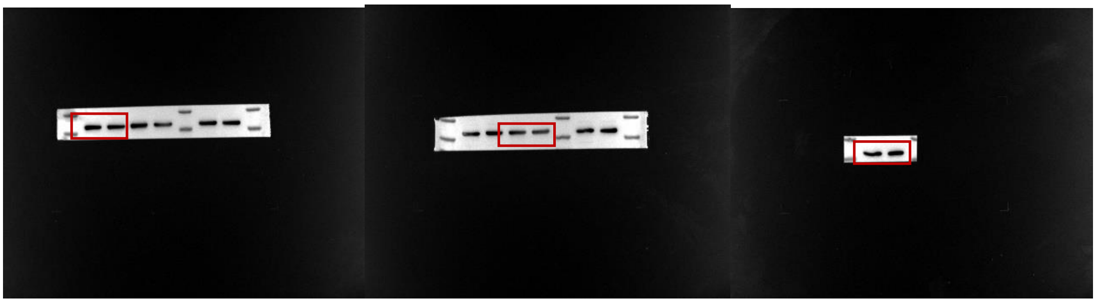

H3K27cr

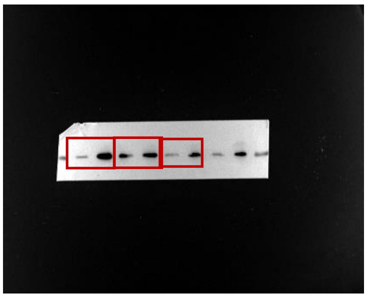

H3

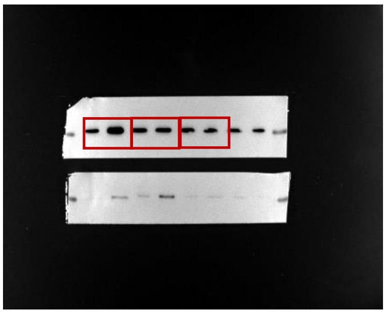

Figure 5D

E-cadherin

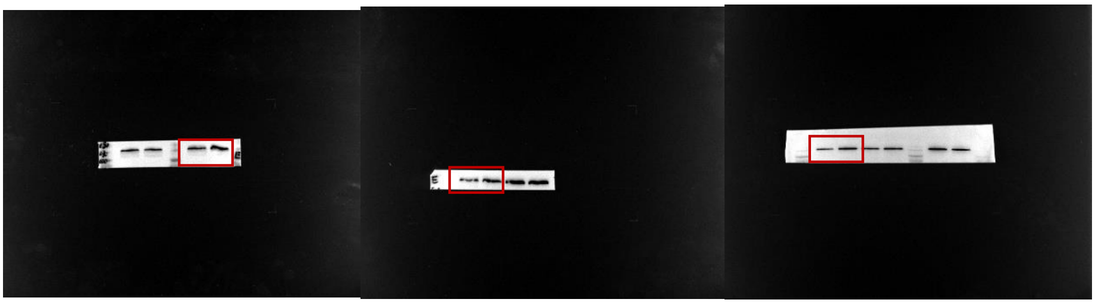

GAPDH

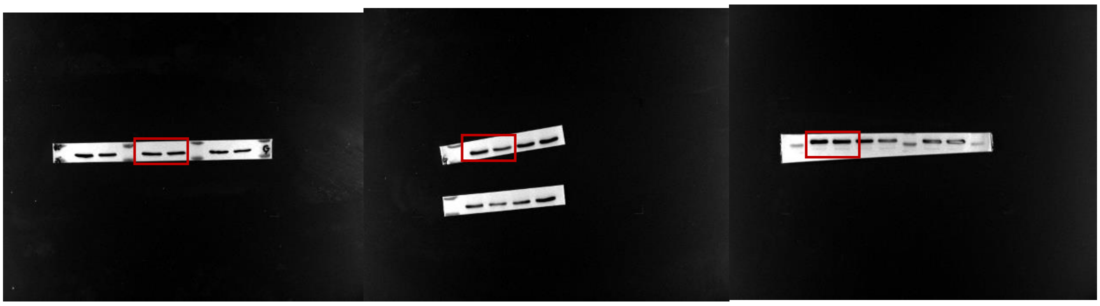

N-cadherin

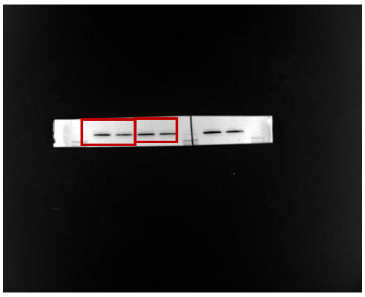

Vimetin

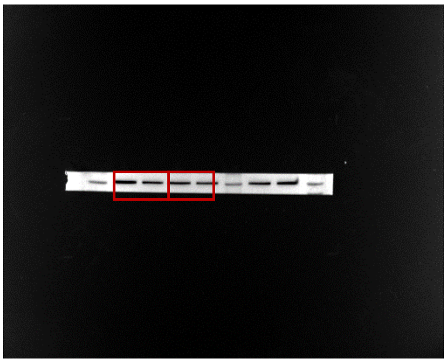

GAPDH

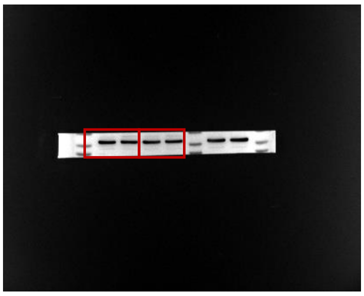

Figure 5E

HCT116

E-cadherin

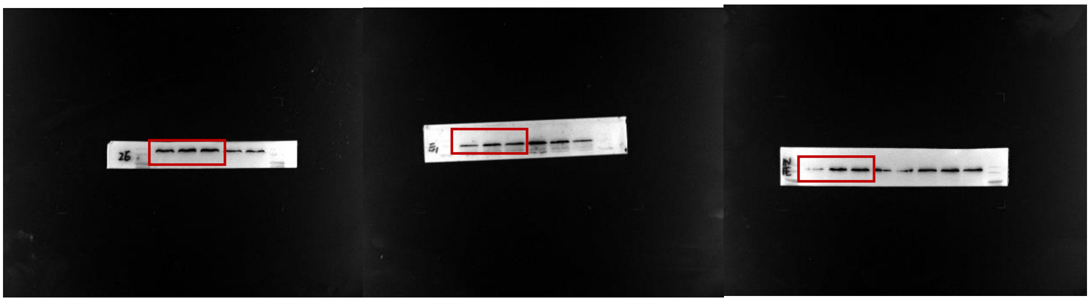

GAPDH

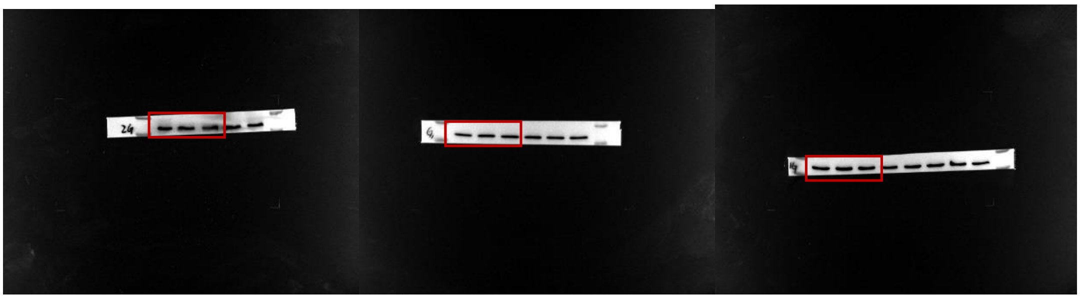

N-cadherin

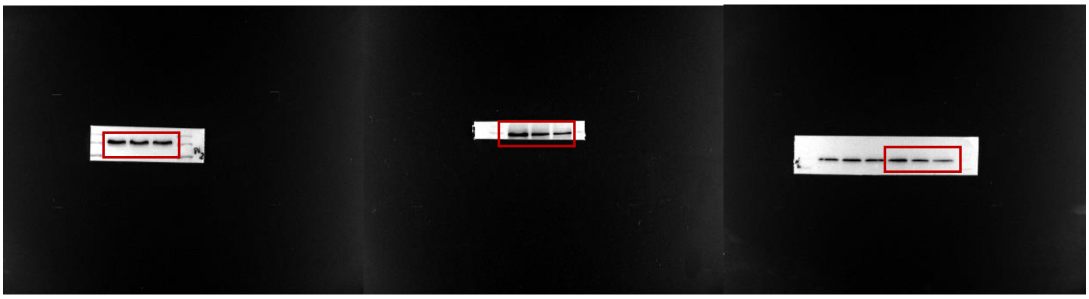

GAPDH

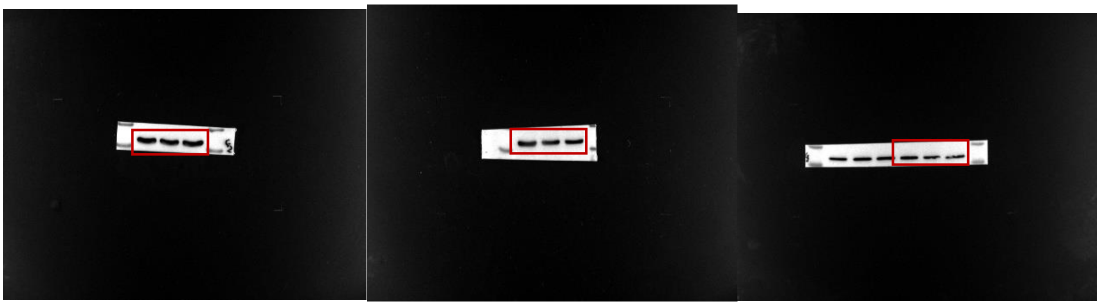

Vimentin

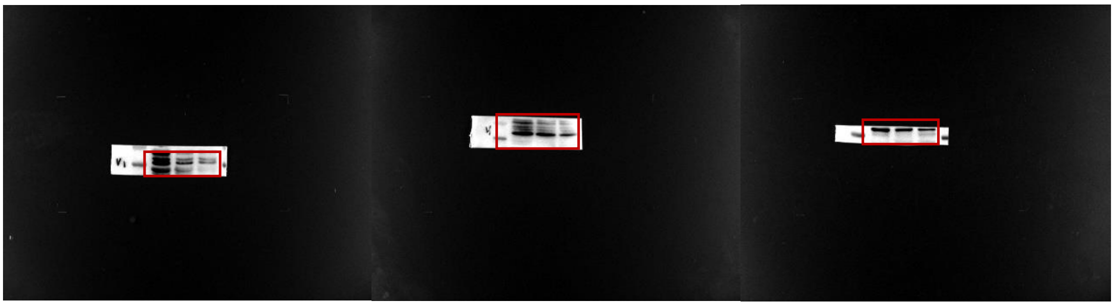

GAPDH

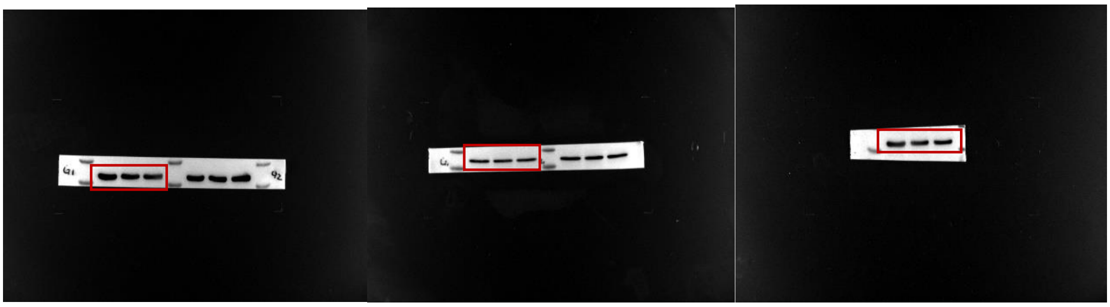

LOVO

E-cadherin

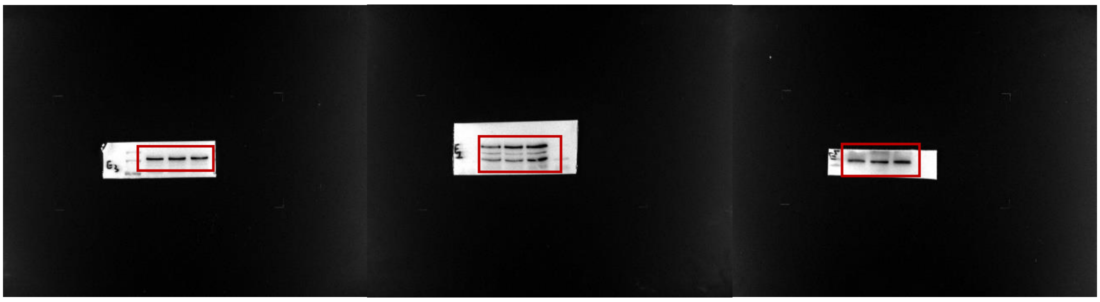

GAPDH

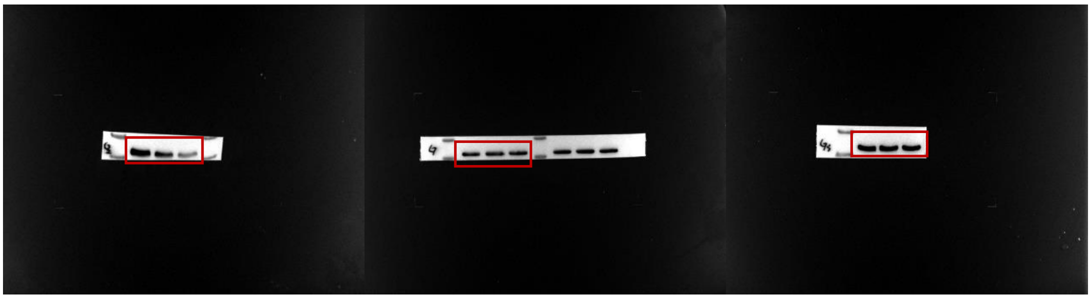

N-cadherin

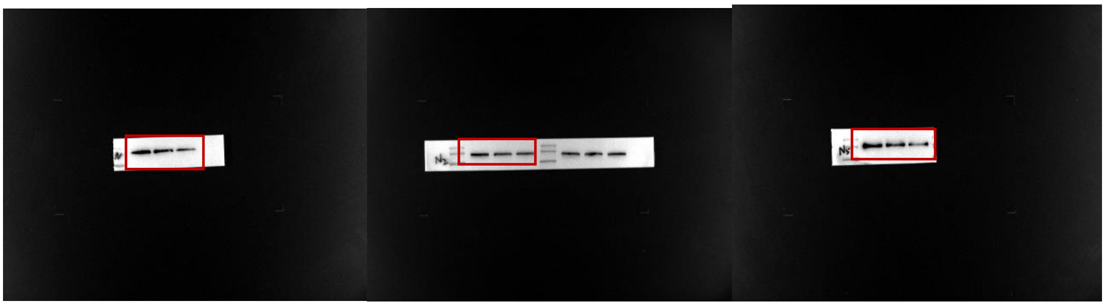

GAPDH

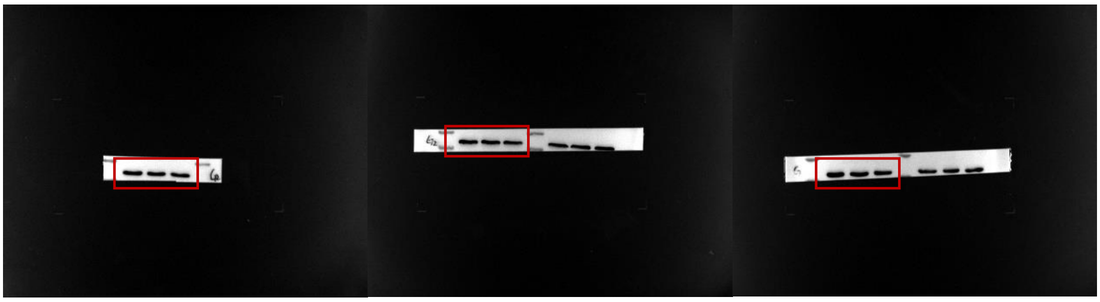

Vimentin

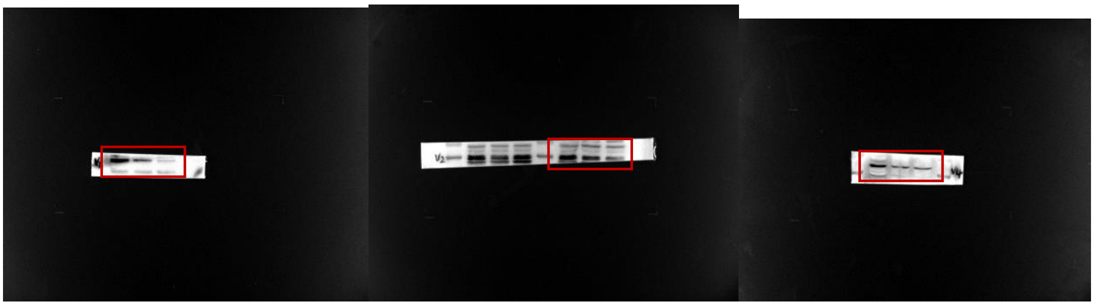

GAPDH

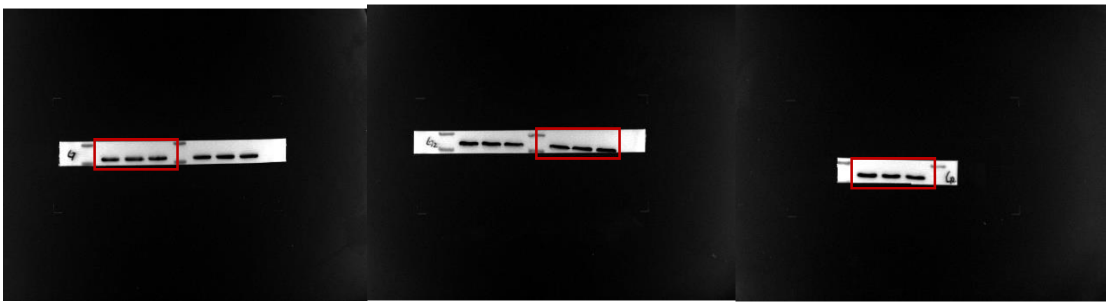

Figure 5F

HCT116

E-cadherin

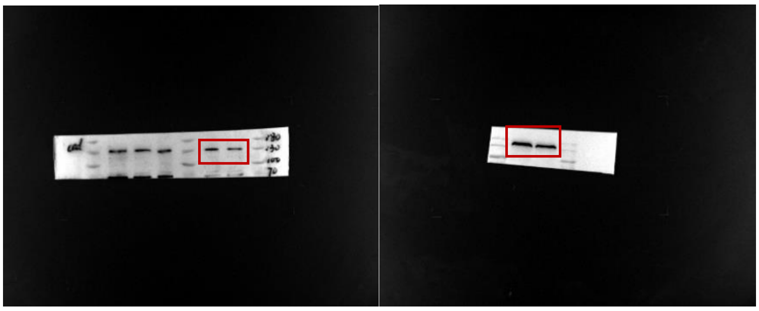

GAPDH

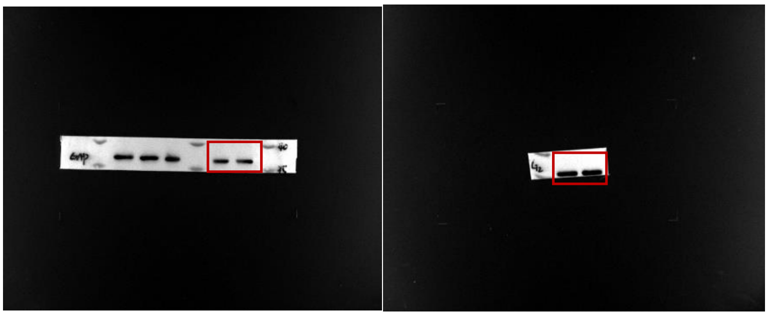

N-cadherin

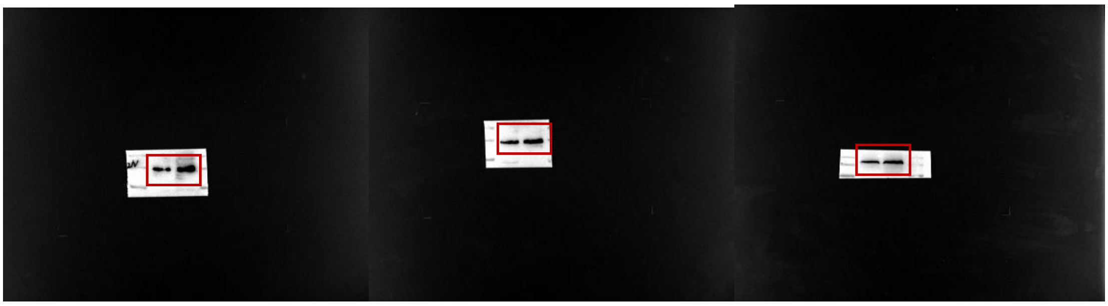

GAPDH

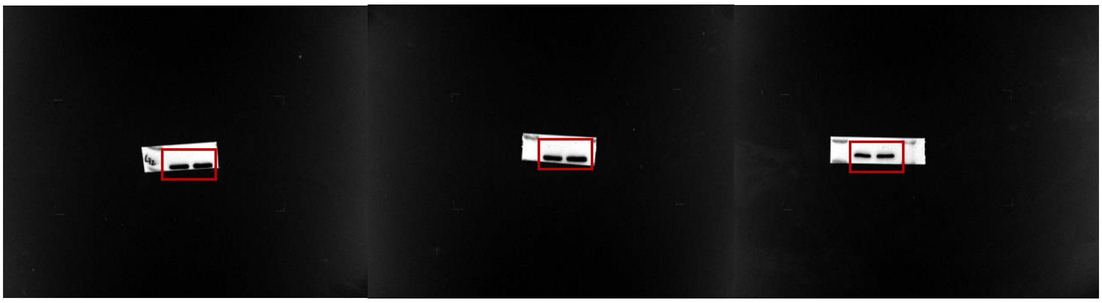

Vimentin

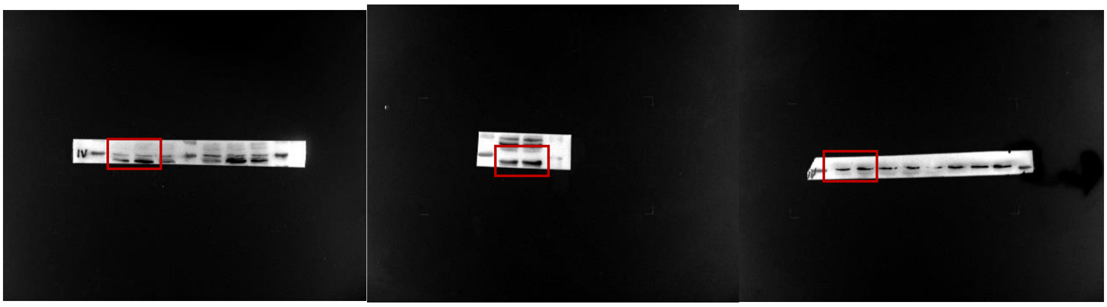

GAPDH

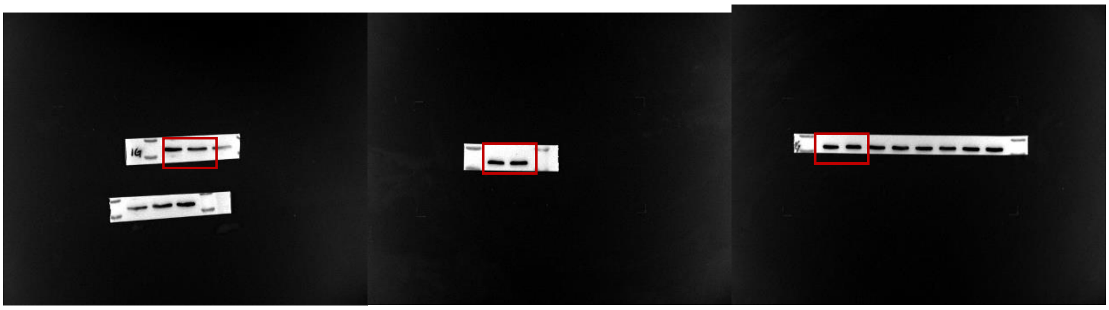

Figure 5F

LOVO

E-cadherin

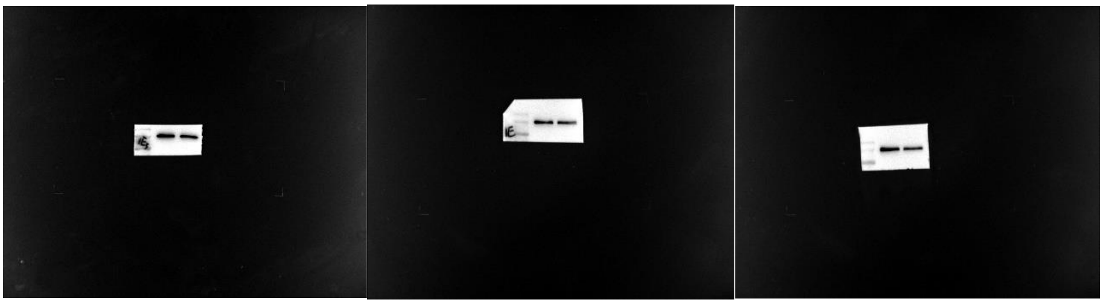

GAPDH

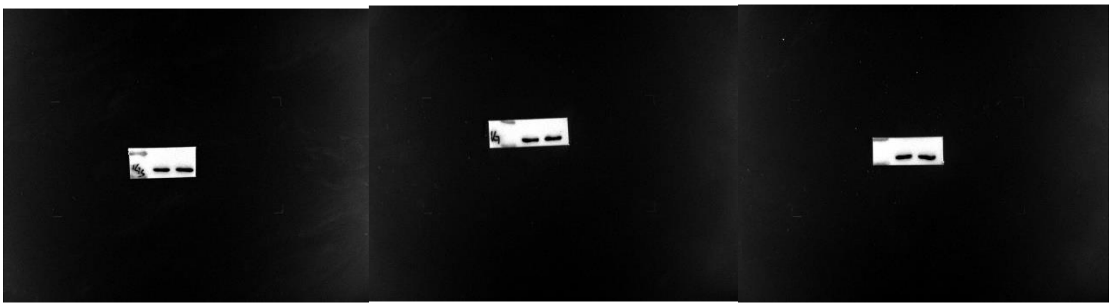

N-cadherin

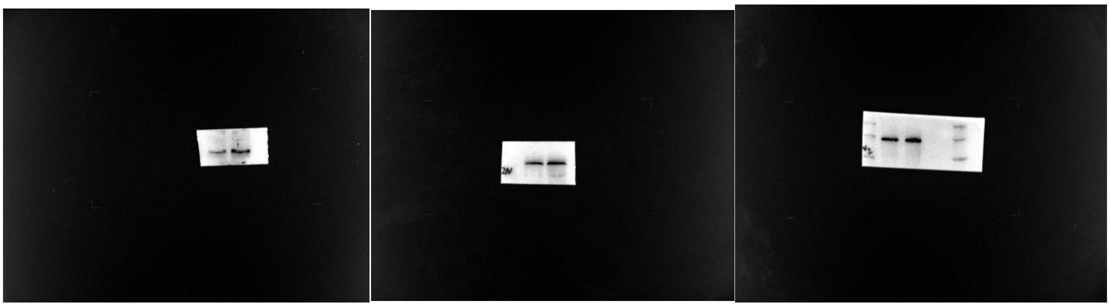

GAPDH

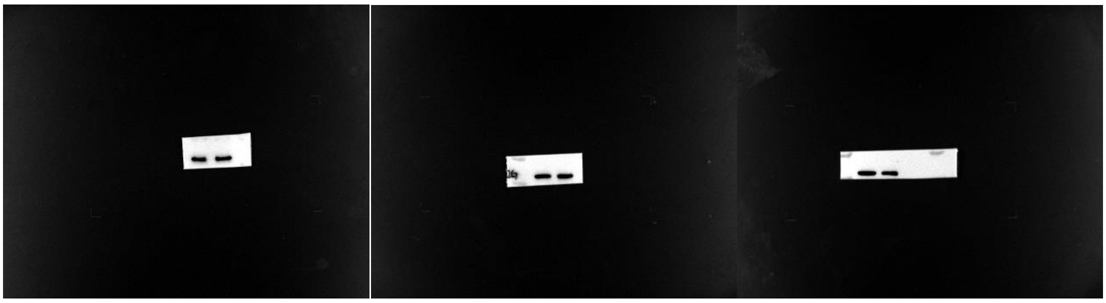

Vimentin

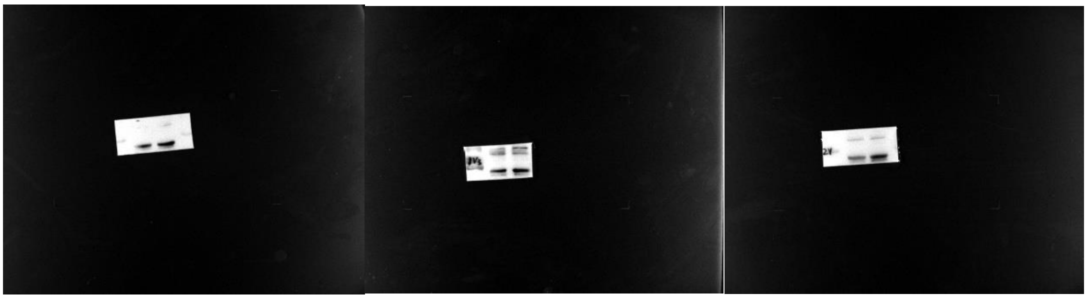

GAPDH

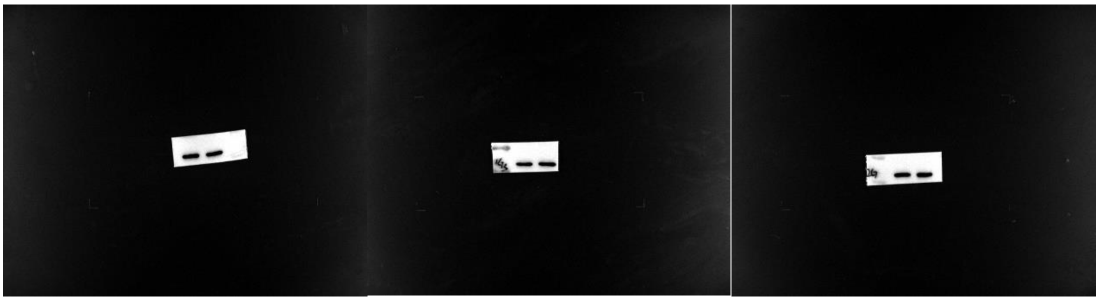

Figure 6B

E-cadherin

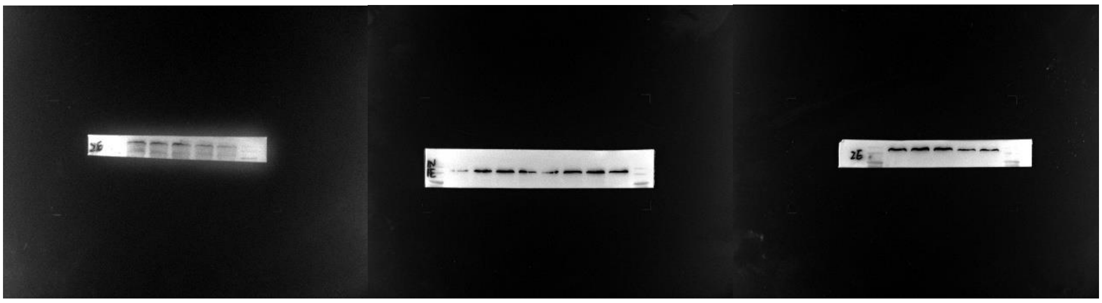

GAPDH

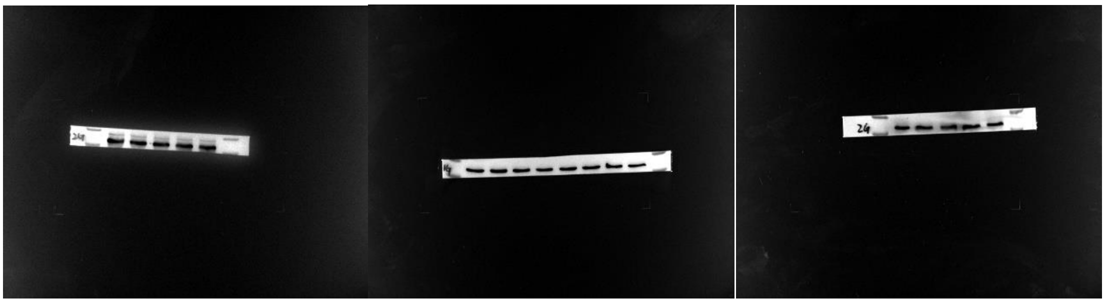

N-cadherin

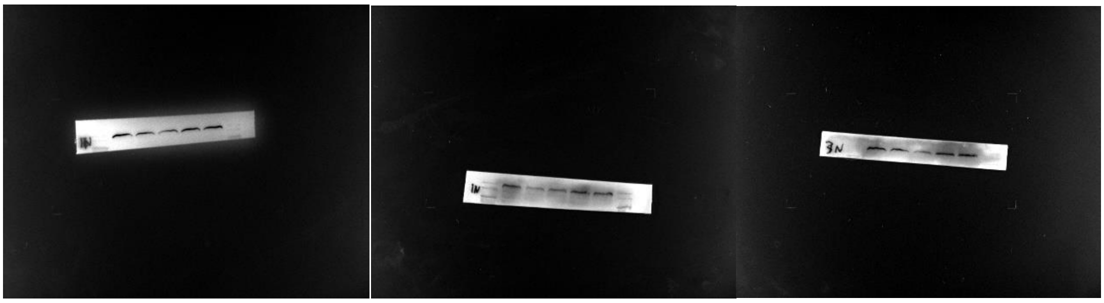

GAPDH

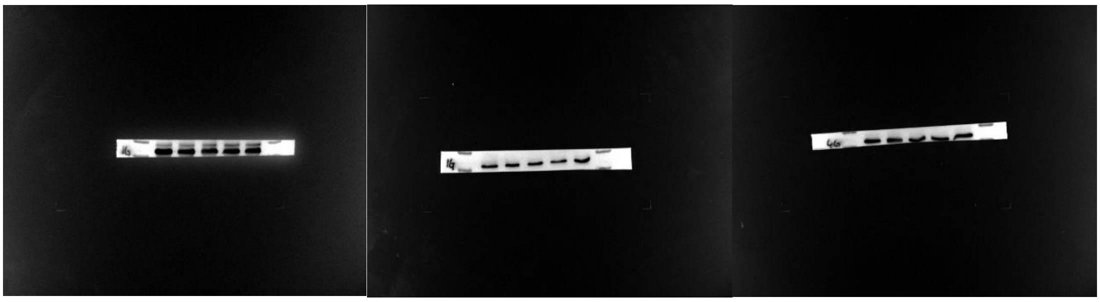

Vimentin

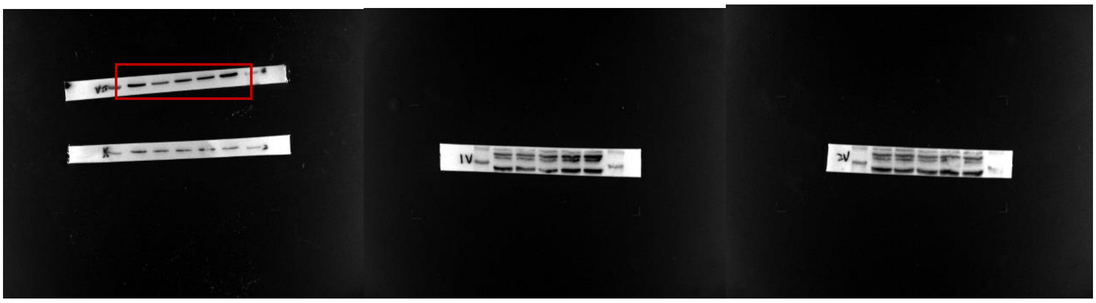

GAPDH

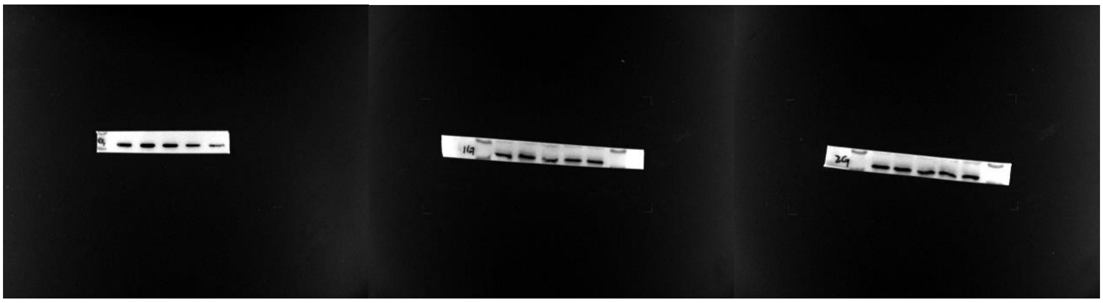

Figure 6C

E-cadherin

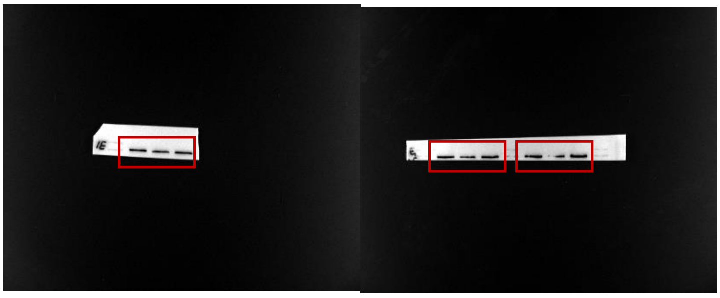

GAPDH

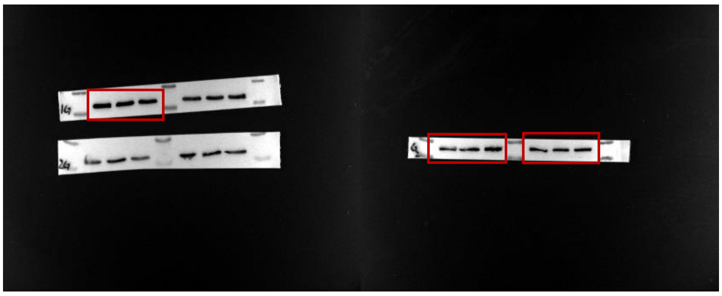

GCN5

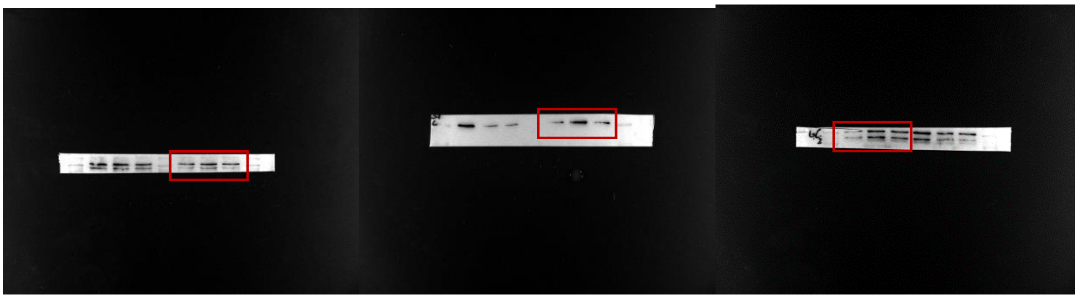

GAPDH

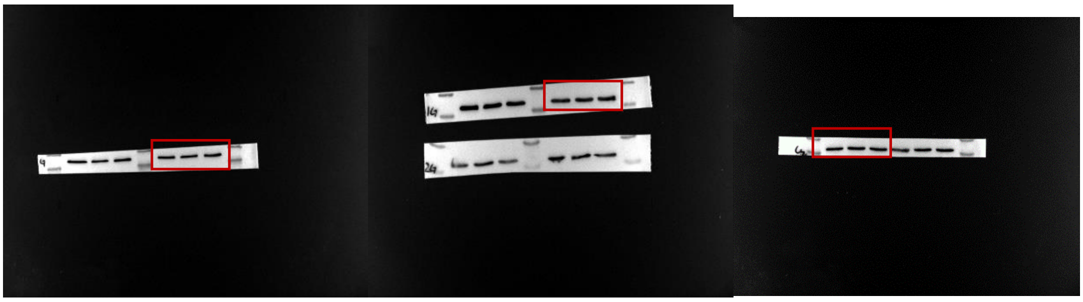

Vimentin

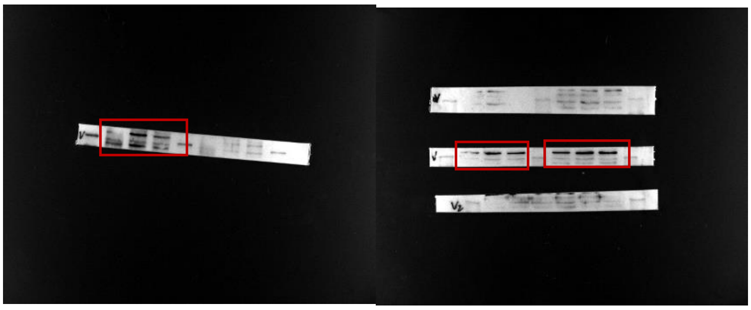

GAPDH

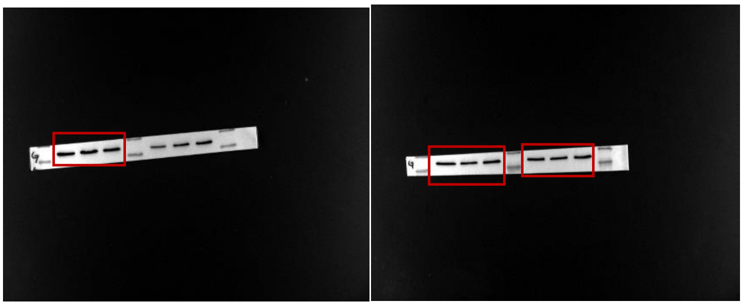

Figure 7C

HCT116

ETS1

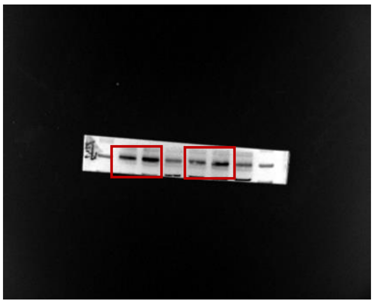

GAPDH

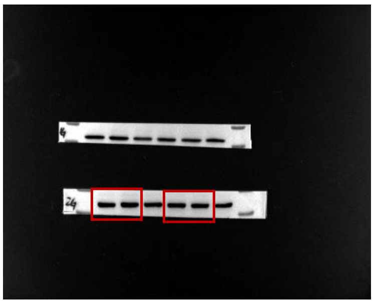

Figure 7C

LOVO

ETS1

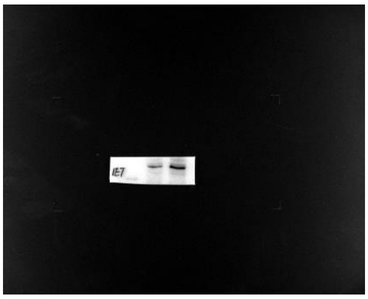

GAPDH

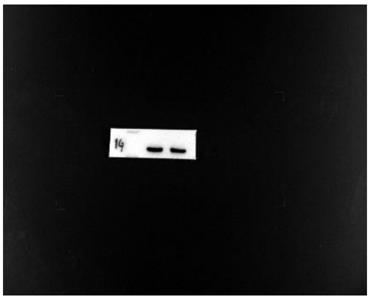

Figure 7D

HCT116

ETS1

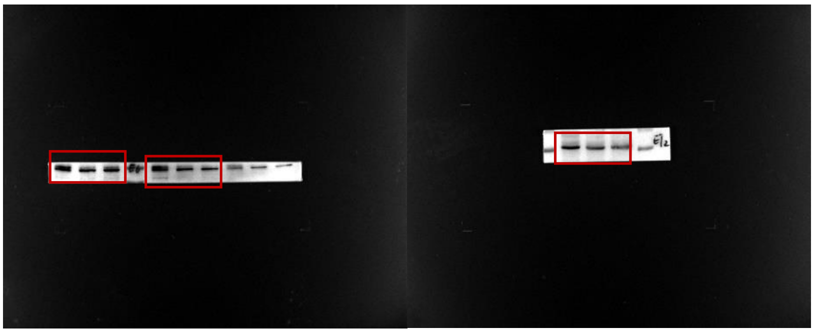

GAPDH

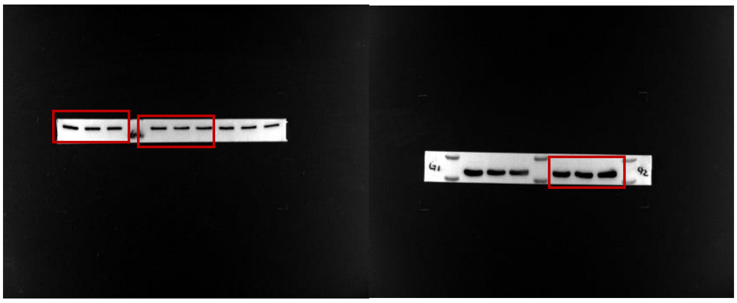

LOVO

ETS1

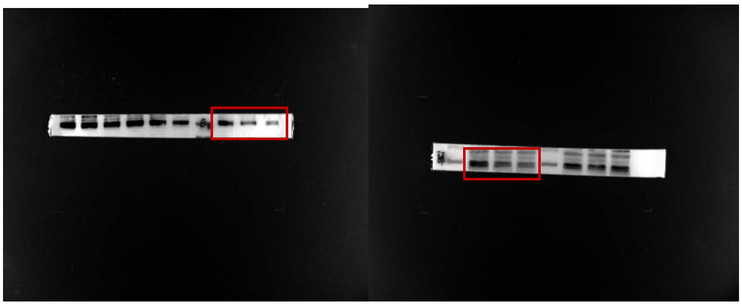

GAPDH

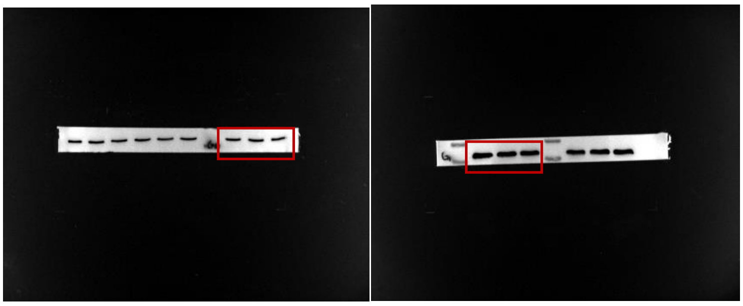

Figure 7F

E-cadherin

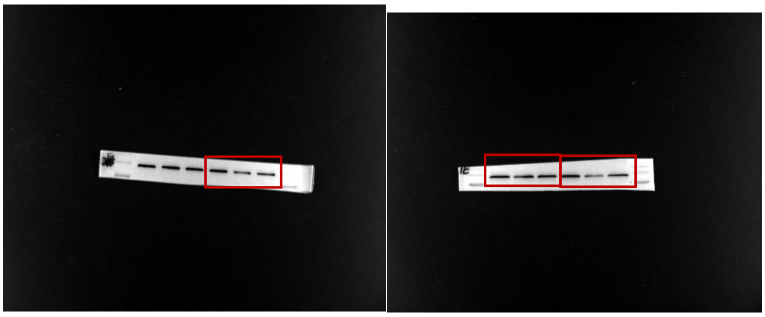

N-cadherin

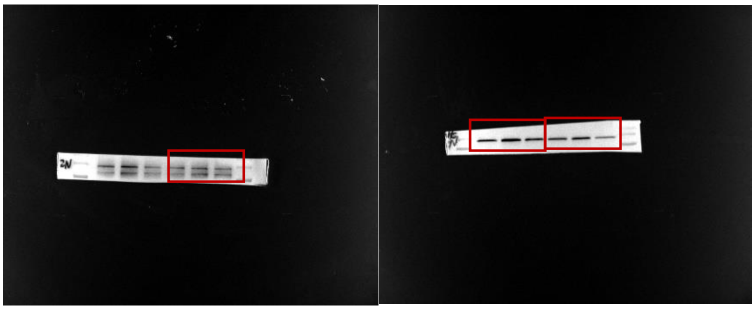

ETS1

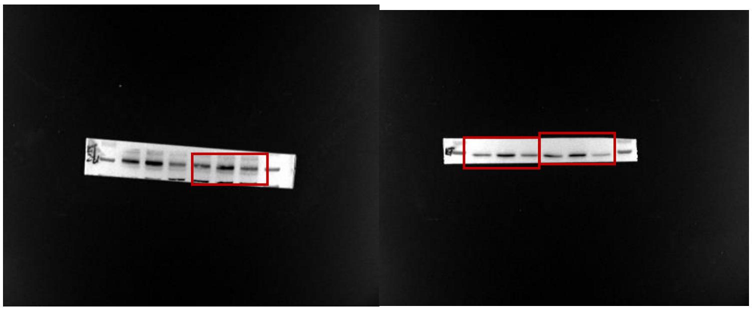

Vimentin

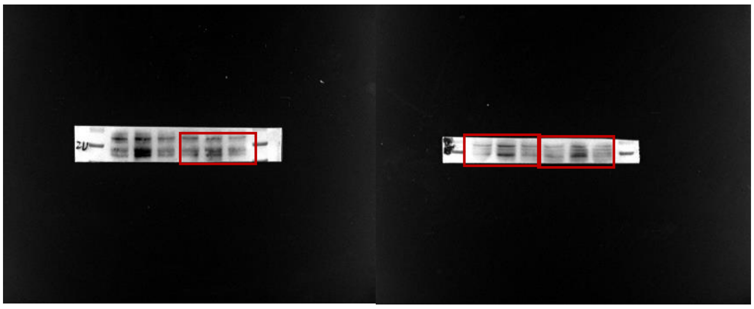

GAPDH

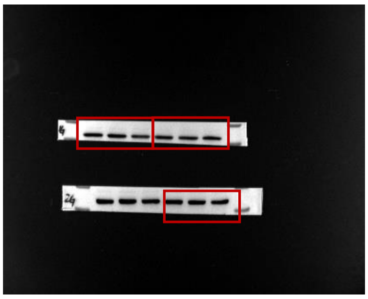

Figure S5A

H3K27cr

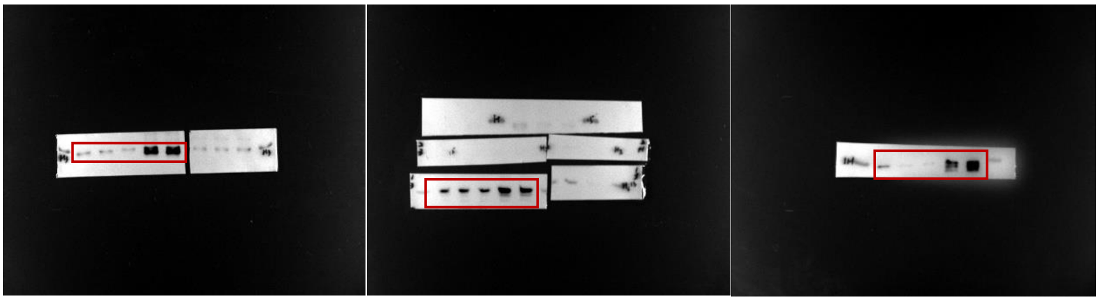

H3

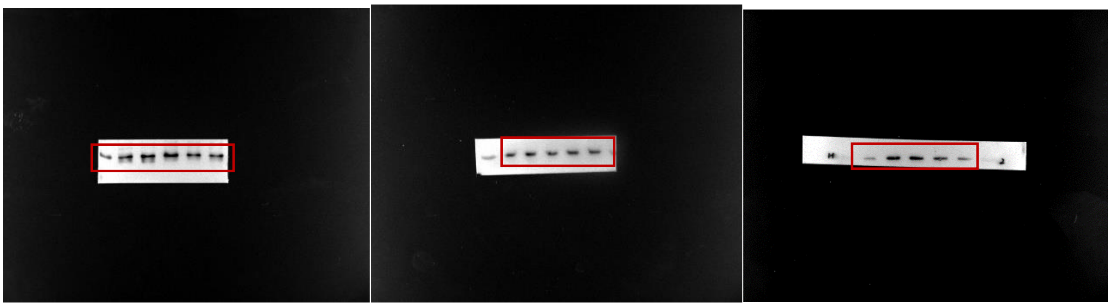

Supplement: Supplementary file 3 — Raw data for Western Blot Experiments [file 41419_2024_7091_MOESM3_ESM.pdf]
